# Adiposity Status Close to Diagnosis and Its Association with Prostate Cancer Survival in the UK Biobank

**DOI:** 10.1158/2767-9764.CRC-25-0124

**Published:** 2025-07-16

**Authors:** Margarita Cariolou, Sofia Christakoudi, Marc J. Gunter, Alicia K. Heath, Amy Berrington de González, Doris S.M. Chan, David C. Muller, Konstantinos K. Tsilidis

**Affiliations:** 1 Department of Epidemiology and Biostatistics, School of Public Health, Faculty of Medicine, Imperial College London, London, United Kingdom.; 2 Division of Genetics and Epidemiology, The Institute of Cancer Research, London, United Kingdom.; 3 Department of Hygiene and Epidemiology, University of Ioannina Medical School, Ioannina, Greece.

## Abstract

Substantial evidence links higher adiposity to prostate cancer development. The relationship between adiposity and outcomes after a prostate cancer diagnosis, however, is unclear. This study aimed to investigate the association between adiposity measured close to prostate cancer diagnosis and all-cause and prostate cancer–specific mortality in a prospective cohort study. Cox regression analyses estimated HRs and 95% confidence intervals (CI) for mortality in 3,760 men in the UK Biobank who had first primary prostate cancer and complete data on body mass index (BMI), waist circumference, hip circumference, waist-to-hip ratio (measured up to 2 years before or up to 5 years after diagnosis), and on covariates (diagnosis age and year, smoking, Townsend deprivation index, exercise, sedentary activities, and alcohol). The waist-to-height ratio and body fat percentage (assessed by bioelectrical impedance) were also evaluated as adiposity measures. Each 5-U increment in pre- or post-diagnosis BMI (*N* = 3,760) was associated with a 30% (95% CI, 1.18–1.44) higher rate of all-cause mortality (deaths = 680), a 33% (95% CI, 1.15–1.52) higher rate of prostate cancer–specific mortality (deaths = 331), and a 28% (95% CI, 1.12–1.47) higher rate of non–prostate cancer mortality (deaths = 347). Positive associations of similar magnitude were observed for separate analyses by pre- and post-diagnosis BMI and for waist and hip circumference, waist-to-hip ratio, waist-to-height ratio, and body fat percentage. Obesity assessed close to prostate cancer diagnosis is associated with higher mortality. More studies are needed to strengthen the evidence and clarify the mechanisms behind the observed associations.

**Significance:**

Patients with prostate cancer might improve their chances of survival by avoiding obesity.

## Introduction

Prostate cancer is a major global health issue. It represents the second most common male cancer and remains a leading cause of cancer-related mortality in men (1). In 2022, there were more than 1.47 million prostate cancer diagnoses and approximately 400,000 prostate cancer–specific deaths (2). Prostate cancer accounts for 28% of all new male cancer diagnoses in the United Kingdom (3). Incidence rates (particularly of low-grade tumors) are expected to increase by 15% between 2023 and 2025 and between 2038 and 2040, equivalent to 85,100 new prostate cancer diagnoses per year (3).

The prognosis of prostate cancer is generally good. Survival rates approach 100% when the tumor is localized (4). Prognosis is poor (around 30%) when the tumor is diagnosed at an advanced stage (4). Most men with prostate cancer survive for at least 5 years after diagnosis (3) and during the past decades, there have been substantial improvements in cancer treatments and survival (5). As a result, individuals living with cancer will continue representing a considerable and fast-growing segment of the population with multiple necessities and challenges that would need to be addressed, including long-term chronic problems, adverse outcomes, and poorer quality of life (6, 7).

Lifestyle behaviors including adherence to a healthy diet, physical activity, and weight management could have an important role in cancer prognosis (6). Currently, there is no lifestyle guidance specifically tailored to patients with prostate cancer. In the absence of targeted lifestyle advice, individuals with cancer are encouraged to follow the cancer prevention recommendations (8, 9). A better understanding of the impact of modifiable factors including adiposity on cancer survival after diagnosis is therefore of paramount importance (8, 10–12).

Adiposity is a relatively well-established modifiable risk factor of prostate cancer, and evidence suggests that the associations differ according to disease aggressiveness (13–17). Higher adiposity has been inversely associated with the risk of developing localized and total prostate cancer (13–15) and positively associated with the risk of developing advanced or fatal prostate cancer (13, 15, 17–19) in studies including the UK Biobank.

The association between adiposity and outcomes after prostate cancer diagnosis, however, is less well understood (8). Observational analyses in men with prostate cancer that investigated adiposity close to or after diagnosis and mortality reported mixed results, mainly based on general adiposity as defined by body mass index (BMI; ref. 12). Few studies incorporated other adiposity indices such as waist and hip circumference. Most observational studies that investigated adiposity close to or after diagnosis in relation to mortality were single-centered, with relatively small sample sizes (12). A recent analysis that we performed in men diagnosed with prostate cancer in the European Prospective Investigation into Cancer and Nutrition cohort identified positive associations between BMI assessed close to diagnosis and mortality outcomes. Results for the other adiposity indices assessed close to diagnosis were less clear compared with BMI due to limited data but suggested positive associations with mortality (20).

The aim of this study was to investigate the associations between adiposity (BMI, waist circumference, hip circumference, waist-to-hip ratio, waist-to-height ratio, and percentage body fat) assessed close to prostate cancer diagnosis in relation to all-cause and prostate cancer–specific mortality in the UK Biobank.

## Materials and Methods

### Study population and characteristics

The UK Biobank (RRID: SCR_012815; ref. 21) is a large population-based prospective study that recruited more than 500,000 individuals (46% men, *N* = 229,114) aged 40 to 70 years across 22 different centers in the United Kingdom i.e., Scotland (7%), Wales (4%), and England (89%). Male individuals were eligible if they had registered with the National Health Service and lived near an assessment center within reasonable traveling distance (up to 25 miles; refs. 22, 23). Between 2006 and 2010, all participants completed baseline questionnaires about lifestyle and sociodemographic characteristics and had objective anthropometric assessments. The percentage of body fat was assessed through bioelectrical impedance for almost all participants (21, 23, 24). Medication use and medical history were recorded. Between 2012 and 2013, 20,000 individuals provided repeat data using questionnaires and measurements including anthropometry. The first imaging visit began after 2014 and aimed to recruit up to 100,000 individuals. Imaging data are currently available from 50,000 individuals. The repeat imaging visit began after 2019 and approximately 70,000 individuals participated. Data from 5,000 repeat images are currently available. Anthropometry data were also collected at the first and repeat imaging visits (25).

### Eligibility criteria

Data from all participating UK centers were used to identify men who had been diagnosed with first primary prostate cancer (prevalent or incident with respect to baseline) using International Classification of Diseases (ICD) version 10 code C61 (ICD-10:C61). The number of men with adiposity data up to 2 years before or up to 5 years after diagnosis was identified for each assessment visit [initial assessment visit (2006–2010) – instance 0, the first repeat assessment visit (2012–2013) – instance 1, the first imaging visit (2014+) – instance 2, and the repeat imaging visit (2019+) – instance 3].

The post-diagnosis exposure window of this study was restricted to 5 years as most prostate cancer recurrences occur within the first 5 years after initial treatment (26–28). The pre-diagnosis exposure window was restricted to 2 years, aiming to collect adiposity data close to diagnosis. Adiposity status in the 2 years until the actual diagnosis date is likely a good representation of the adiposity status at diagnosis and probably not affected by the tumor itself as most prostate cancer tumors are diagnosed at early stages while they are still asymptomatic (29).

Individuals with prevalent prostate cancer who had a diagnosis of first primary prostate cancer up to 5 years before the date of the initial assessment visit were eligible for this study. The pre-diagnosis adiposity assessment analyses therefore included men with incident prostate cancer who (i) either had baseline or (ii) follow-up data (from any repeat assessment) collected up to 2 years before diagnosis. The post-diagnosis analyses included men with prevalent prostate cancer at study entry who had baseline data collected up to 5 years after diagnosis or (ii) men with incident prostate cancer who had follow-up data collected up to 5 years after diagnosis from any repeat assessment. For each participant, the first available adiposity measurement close to diagnosis was taken; therefore, there was no overlap of individuals in each assessment period.

### Adiposity variables

All adiposity indices were measured by trained staff following established protocols (including all men in our study with prevalent or incident prostate cancer). Waist circumference was measured at the natural indent, or the umbilicus, and hip circumference was measured at the widest point. BMI was calculated by dividing weight (kg) by the square of standing height (m^2^; ref. 30). A waist-to-hip ratio was calculated as the ratio of waist circumference divided by hip circumference. A waist-to-height ratio was calculated as the ratio of waist circumference divided by standing height. BMI was also classified according to the World Health Organization (WHO) categories as underweight <18.5 kg/m^2^, normal weight 18.5 to 24.9 kg/m^2^, overweight 25 to 29.9 kg/m^2^, and obese ≥30 kg/m^2^. Body fat percentage was assessed by the Tanita BC418MA body composition analyzer.

### Covariates

Based on subject matter knowledge, models were adjusted for relevant covariates selected *a priori* namely age and year of diagnosis, smoking status, physical activity, sedentary behavior, Townsend deprivation index as proxy for socioeconomic status, and alcohol consumption. Supplementary Table S1 and the directed acyclic graph in Supplementary Fig. S1 present assumptions of potential causal relations between the variables of the present study, considering evidence from the literature.

Complete case analyses were performed (i.e., the analytic sample was restricted to individuals with complete information on the covariates; ref. 31).

Self-reported physical activity was evaluated through an adapted version of the International Physical Activity Questionnaire completed on a tablet computer (32). Participants were asked to report on the number of days they engaged in more than 10 minutes of each: walking, moderate physical activity, and vigorous physical activity in a usual week. If participants gave an answer of at least 1 day, they were questioned for the minutes they engaged in each of the activities on a typical day. Physical activity was included in models in metabolic equivalent of task (MET)-hours/week. One MET is expended by sitting quietly for 1 hour, and the MET value reflects the ratio of energy expended per kilogram of body weight per hour to that expended when sitting quietly (33–36). To obtain MET-hours/week, procedures by Bradbury and colleagues (37) were followed. Time spent in sedentary activities was calculated in hours per day as total time spent watching television, using a computer screen, or driving. Men with implausible values of >23 hours per day were excluded from the analysis (38). Smoking status was defined as never, previous, or current (self-reported at the point of recruitment; ref. 39). Townsend deprivation index represents a score that assigns individuals based on the output area (smallest UK census area) in which their postcode was located and was used as quintiles in the models. Higher scores indicated greater socioeconomic deprivation (40). Alcohol intake frequency was assessed in the touchscreen questionnaire defined as never, special occasions only, one to three times monthly, once or twice weekly, three or four times weekly, or daily or almost daily (41, 42).

### Statistical analysis

Cox proportional hazards regression models were used among men with a diagnosis of first primary prostate cancer to estimate HRs and 95% confidence intervals (95% CI) for the associations between adiposity indices assessed close to diagnosis (either up to 2 years before diagnosis or up to 5 years after diagnosis) in relation to all-cause, prostate cancer–specific, and non–prostate cancer mortality. Analyses were performed for each time frame individually and by combining both timeframes (i.e., having in one model all individuals with adiposity data close to diagnosis – either had pre- or post-diagnosis adiposity data). The date of each respective assessment visit (according to the period that the individual entered the study) was considered as the start of follow-up (entry time). The date of death or the date of censoring (December 31, 2020) was considered as the end of follow-up (exit time). The underlying cause of death was coded using ICD-10:C61 and was used to identify deaths from prostate cancer–specific mortality. For the analysis of prostate cancer–specific mortality, deaths from other causes (deaths apart from prostate cancer) were censored. For the analyses of non–prostate cancer mortality, prostate cancer–specific deaths were censored. The Fine–Gray method was used to calculate sub-distribution HRs for prostate cancer–specific and non–prostate cancer mortality to account for competing causes of death (43, 44).

We obtained estimates of the linear associations between adiposity variables and mortality per 5 kg/m^2^ increments in BMI, 10 cm increments in waist and hip circumference, and 0.1-U increments in the waist-to-hip-ratio. Restricted cubic splines were used to investigate possible nonlinear associations, with 3 knots at the 10th, 50th, and 90th percentiles of the adiposity variable distribution (45). The median value of each adiposity variable in each analysis was used as the referent.

Multivariable models were adjusted for the relevant covariates selected a *priori* as described. The minimally adjusted model was adjusted for age and the year of diagnosis, and the fully adjusted model was additionally adjusted for smoking status, physical activity (MET-hours/week), sedentary activities (hours/day), Townsend deprivation index, and alcohol intake frequency. Models were stratified by the UK Biobank center. The proportional hazards assumption was assessed via graphical inspection of the smoothed scaled Schoenfeld residuals (46). Graphical inspection of the smoothed scaled Schoenfeld residuals did not show evidence of violation of the proportional hazards assumption.

### Sensitivity analyses

Lagged analysis was performed to assess possible reverse causation. Because of the lack of data on stage and grade, an analysis excluding men with advanced (including metastatic) prostate cancer was not possible. To more appropriately define the period of follow-up that should be excluded in the lagged analysis, the literature was searched to identify the most representative survival time (average/median) of individuals with advanced prostate cancer. According to literature, the most representative period to be excluded was one to 2 years (47–49). Analyses were therefore performed excluding the first year of follow-up and in a separate analysis excluding the first 2 years of follow-up after the adiposity assessment. Analyses by the year of adiposity measurement were performed for each adiposity index (i.e., at 1 and 2 years before diagnosis and for each year after diagnosis). Each year might reflect differences across the cancer survivorship trajectory, e.g., there might be exposure fluctuations close to/during treatment (50).

There were very few men with repeated adiposity data (i.e., measured in more than one assessment visit according to the eligibility criteria of the present study); therefore, time-varying associations could not be investigated. Analyses were also conducted according to the WHO BMI categories and across subgroups of smoking status. In addition, sensitivity analyses were conducted to investigate whether the associations including prevalent versus incident prostate cancers differ.

To check for potential selection bias, we compared important baseline lifestyle characteristics of the men with prostate cancer who had data on all the adiposity indices (BMI, waist circumference, hip circumference, and waist-to-hip ratio) and complete information on the covariates of the main model according to the eligibility criteria of the present study (*N* = 3,760) with those who did not (*N* = 4,859). We also compared the lifestyle characteristics of the men who were included in the separate pre- (*N* = 1,390) and post-diagnosis analyses (*N* = 2,370).

The possibility of performing an analysis using dual-energy X-ray absorptiometry, as a more objective measure of assessing body composition, was explored (51), but limited data were available according to the eligibility criteria of the present study.

RStudio version 4.0.5 (RRID: SCR_000432) was used for analyses.

### Data availability

The dataset analyzed in the current study can be requested from the UK Biobank. For information on sending application to gain access to UK Biobank data, please follow the instructions at https://www.ukbiobank.ac.uk/enable-your-research.

### Ethics approval and consent to participate

This research was conducted according to the principles expressed in the Declaration of Helsinki. The UK Biobank cohort has been approved by the North West Multicenter Research Ethics Committee, United Kingdom (ref: 16/NW/0274). Written informed consent has been obtained from all study participants. The current study was approved by the UK Biobank access management board. Participants who had withdrawn consent by the time of the analysis were excluded from the analysis dataset.

## Results

### Cohort characteristics

**Figure 1:**
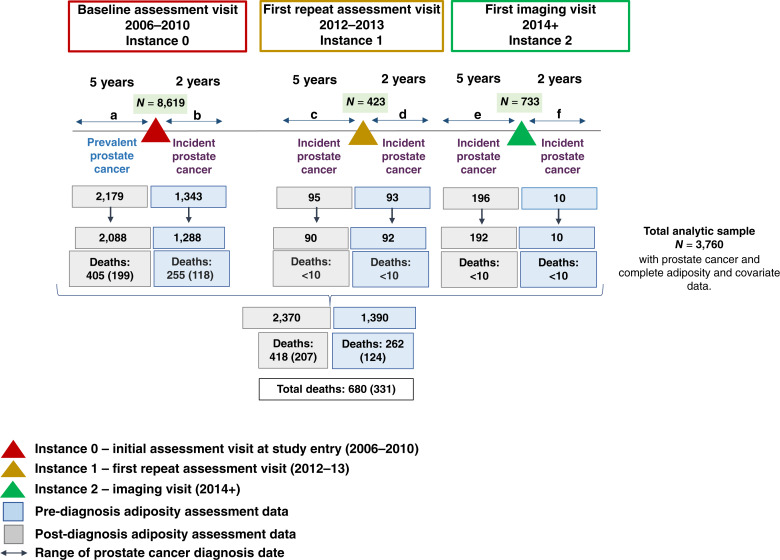
Data collection time frames for adiposity data – study design. The baseline assessment visit, in which adiposity measurements were taken, occurred between 2006 and 2010 (instance 0). Adiposity measurements including body fat percentage by bioelectrical impedance were also taken from some individuals in the first repeat assessment visit that occurred between 2012 and 2013 (instance 1) and in the first imaging visit after 2014 (instance 2). The pre-diagnosis analyses (blue boxes) included men with incident prostate cancer who (1) either had baseline or (2) follow-up adiposity data (from any repeat assessment) collected up to 2 years before diagnosis. The post-diagnosis analyses (gray boxes) include (1) men with prevalent prostate cancer at study entry who had baseline data collected up to 5 years after diagnosis or (2) men with incident prostate cancer who had follow-up data collected up to 5 years after diagnosis (from any repeat assessment). Green boxes indicate the number of men with prostate cancer who participated in each assessment visit. Deaths: all-cause (prostate cancer specific). Each timeframe/period (a–f) excludes individuals from previous time frames/periods (a–f) as appropriate (no overlap of individuals in the final analytic sample for each adiposity index). The final analytic sample of the present study is those who had adiposity data on BMI, waist and hip circumference, and waist-to-hip ratio and complete data on the covariates of the main model [age of diagnosis, year of diagnosis, smoking status, physical activity, sedentary activities (sum of time spent watching TV, using a computer screen, or driving in hours/day), Townsend deprivation index (quintiles), and alcohol intake frequency. The person years of observation in the pre- or post-diagnosis analysis were 38,551; in the post-diagnosis analysis, they were 23,937, and in the pre-diagnosis analysis, they were 14,614 (rounded to the nearest whole number)].

A total of 8,619 men had a first primary prostate cancer diagnosis (ICD-10: C61, either prevalent at study entry or incident prostate cancer during cohort follow-up). According to the eligibility criteria of this study, 3,916 men had data on BMI, waist circumference, and hip circumference collected close to prostate cancer diagnosis. Missing data from the covariates utilized in the main model are shown in Supplementary Table S2. Of the 3,916 men, 3,760 had complete covariate data and represented the analytic sample of the present study. Of the 3,760 men, 2,088 (56%) had prostate cancer at study entry (prevalent; Fig. 1).

Important demographic and lifestyle characteristics of the men are shown in Table 1. Men were diagnosed between 2001 and 2016 and the range of age at diagnosis was 40 to 77 years. The median BMI of all the individuals with prostate cancer and adiposity data was 27.3 kg/m^2^. Most men were never (47%) or former smokers (44%) and few (8%) were current smokers. The majority (50%) were involved in moderate intensity physical activity and consumed alcohol once or twice weekly (25%), three or four times weekly (23%), or daily or almost daily (29%).

**Table 1:** Lifestyle characteristics of the 3,760 men diagnosed with prostate cancer who had adiposity data in the UK Biobank

Characteristic	Pre- or post-diagnosis adiposity data *N* = 3,760	Post-diagnosis adiposity *N* = 2,370	Pre-diagnosis adiposity *N* = 1,390
Follow-up time i.e., from the baseline or follow-up questionnaire/assessment until censoring/death, median years (p2.5–p97.5)	11.4 (1.85–13.3)	11.4 (1.67–13.3)	11.4 (2.45–13.3)
Age at diagnosis, years [median (p2.5–p97.5)]	64 (52–71)	63 (52–70)	66 (54–72)
Age at diagnosis, years (range)	40–77	40–77	43–76
Year of diagnosis, range	2001–2016	2001–2016	2007–2016
BMI (kg/m^2^)	​	​	​
Median (p2.5–p97.5)	27.3 (21–36)	27.4 (21–36)	27.1 (21–36)
Range	16.5–51.7	17.7–51.7	16.5–51.0
Normal weight (≤24.9 kg/m^2^), *n* (%)**^a^**	926 (25)	566 (24)	360 (26)
Overweight (25–29.9 kg/m^2^), *n* (%)	1,938 (52)	1,215 (51)	723 (52)
Obese (≥30 kg/m^2^), *n* (%)	896 (24)	589 (25)	307 (22)
Waist circumference (cm)	​	​	​
Median (p2.5–p97.5)	97 (79–120)	97 (79–120)	97 (66–150)
Range	66–158	68–158	66–150
Hip circumference (cm)	​	​	​
Median (p2.5–p97.5)	103 (91–118)	103 (91–118)	103 (91–118)
Range	79–156	82–103	82–156
Waist-to-hip ratio	​	​	​
Median (p2.5–p97.5)	0.94 (0.82–1.07)	0.94 (0.82–1.08)	0.94 (0.82–1.07)
Range	0.69–1.21	0.69–1.21	0.73–1.18
Smoking status	​	​	​
Never smoker, *n* (%)	1,788 (47)	1,154 (49)	634 (46)
Previous smoker, *n* (%)	1,688 (44)	1,049 (44)	639 (46)
Current smoker, *n* (%)	284 (8)	167 (7)	117 (8)
Physical activity (sum in excess MET-hours/week)^b^	​	​	​
Low activity <10 excess MET-hours/week, *n* (%)	1,106 (29)	703 (30)	403 (29)
Moderate activity 10–49.9 MET-hours/week, *n* (%)	1,868 (50)	1,166 (49)	702 (51)
High activity ≥50 excess MET-hours/week, *n* (%)	786 (21)	501 (21)	285 (21)
Sedentary activities (hours/day)^c^	​	​	​
Range	0–21	0–17	0–21
Median (p2.5–p97.5)	5 (2–11)	5 (2–11)	5 (2–11)
Townsend deprivation index^d^	​	​	​
Range	–6.3 to 8.9	–6.3 to 8.6	–6.18 to 8.89
Median (p2.5–p97.5)	–2.5 (–5.5 to 5.6)	–2.5 (–5.7 to 5.7)	–2.5 (–5.4 to 5.6)
In quintiles	​	​	​
Quintile 1 – least deprived	755 (20)	474 (20)	278 (20)
Quintile 2	749 (20)	475 (20)	279 (20)
Quintile 3	752 (20)	473 (20)	277 (20)
Quintile 4	752 (20)	474 (20)	278 (20)
Quintile 5 – most deprived	752 (20)	474 (20)	278 (20)
Alcohol intake frequency	​	​	​
Never, *n* (%)	228 (6)	159 (7)	69 (5)
Special occasions only, *n* (%)	293 (8)	192 (8)	101 (7)
One to three times monthly, *n* (%)	312 (9)	216 (9)	96 (7)
Once or twice weekly, *n* (%)	923 (25)	585 (25)	338 (24)
Three or four times weekly, *n* (%)	951 (23)	597 (25)	354 (25)
Daily or almost daily, *n* (%)	1,053 (29)	621 (26)	432 (31)
Ethnicity^e^	​	​	​
White, *n* (%)	3,602 (96)	2,275 (96)	1,327 (95)
Other, *n* (%)	158 (4)	95 (4)	63 (5)

Percentages are rounded to the nearest whole number and may therefore not add up to 100. The characteristics of individuals are taken from each respective time frame (either baseline or follow-up) that the individual was selected from according to the eligibility criteria of the present study.

a The normal weight category includes the underweight as the number of men with underweight was very small (<10 men).

b Physical activity as the sum of walking and moderate and vigorous activities in excess MET-hours/week.

c Sedentary activities as the sum of total time spent watching television, using a computer screen, or driving in hours/day.

d Townsend deprivation index quintiles for the pre- and post-diagnosis adiposity combined (full dataset, *N* = 3,760): −6.26 to −4.08, −4.08 to −3.02, −3.02 to −1.82, −1.82 to 0.652, and 0.652–8.89; for the post-diagnosis adiposity subset, *N* = 2,370: −6.26 to –4.03, −4.03 to −2.99, −2.99 to −1.81, −1.81 to 0.772, and 0.772–8.59]; and for the pre-diagnosis adiposity subset: −6.18 to −4.16, −4.16 to −3.08, −3.08 to −1.84, −1.84 to 0.508, and 0.508–8.89.

e Ethnicity: ^**“**^White” includes White, British, Irish, and any other White background; “Other” includes mixed, Asian or Asian British, Black or Black British, Chinese, other ethnic group, and those who did not know/prefer not to answer.

Most men were White (96%; Table 1). Important lifestyle characteristics of the 3,760 men were largely similar according to the WHO BMI categories. Obese men were more likely to be previous smokers (52%) whereas the group of under- and normal weight men was more likely to be never smokers (56%). A similar proportion of current smokers was observed between obesity categories (Supplementary Table S3).

The 3,760 men had adiposity data collected either up to 2 years before (*N* = 1,390) or up to 5 years after diagnosis (*N* = 2,370; Fig. 1) and were followed for a median of 11.4 years from the baseline or follow-up questionnaire/assessment (Table 1).

### Main results

#### BMI

**Figure 2:**
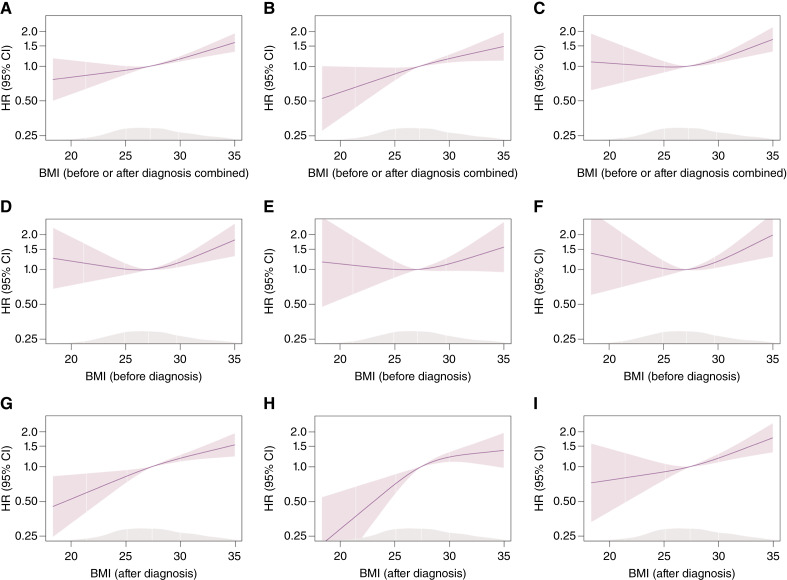
Restricted cubic spline analysis for BMI assessed close to diagnosis and all-cause, prostate cancer–specific, and non–prostate cancer mortality. HRs from the Cox proportional hazards model with restricted cubic spline curves describing the association between BMI (kg/m^2^) collected before or after diagnosis combined and (**A**) all-cause mortality (deaths = 680), (**B**) prostate cancer–specific mortality (deaths = 331), and (**C**) non–prostate cancer mortality (deaths = 347); pre-diagnosis BMI and (**D**) all-cause mortality (deaths = 262), (**E**) prostate cancer–specific mortality (deaths = 124), and (**F**) non–prostate cancer mortality (deaths = 137); and post-diagnosis BMI and (**G**) all-cause mortality (deaths = 418; **H**), prostate cancer–specific mortality (deaths = 207), and (**I**) non–prostate cancer mortality (deaths = 210). HRs are based on the main model adjusted for age of diagnosis, year of diagnosis, smoking status, physical activity, sedentary activities, Townsend deprivation index, and alcohol intake frequency; knots at the 10th, 50th, and 90th percentiles of BMI. The median BMI of the individuals included in analyses was used as referent: 27.3 kg/m^2^ in the pre- or post-diagnosis analysis, 27.1 kg/m^2^ in the pre-diagnosis analysis, and 27.4 kg/m^2^ in the post-diagnosis analysis. The smooth density plot represents the density of the population across the spline variable.

Analysis of BMI assessed before or after diagnosis combined showed a linear increase in the rate of all-cause (HR per 5 kg/m^2^ = 1.30; 95% CI, 1.18–1.44), prostate cancer–specific (HR per 5 kg/m^2^ = 1.33; 95% CI, 1.15–1.52), and non–prostate cancer mortality (HR per 5 kg/m^2^ = 1.28; 95% CI, 1.12–1.47). Positive associations were observed both for pre-diagnosis BMI and all-cause (HR per 5 kg/m^2^ = 1.25; 95% CI, 1.05–1.48), prostate cancer–specific (HR per 5 kg/m^2^ = 1.18; 95% CI, 0.92–1.51), and non–prostate cancer mortality (HR per 5 kg/m^2^ = 1.29; 95% CI, 1.02–1.63) and for post-diagnosis BMI and all-cause (HR per 5 kg/m^2^ = 1.37; 95% CI, 1.21–1.54), prostate cancer–specific (HR per 5 kg/m^2^ = 1.43; 95% CI, 1.20–1.69), and non–prostate cancer mortality (HR per 5 kg/m^2^ = 1.31; 95% CI, 1.11–1.56) as shown in Tables 2–4 and Fig. 2. Inference from the results of the categorical analyses was similar (Supplementary Table S4).

**Table 2:** Cox proportional HRs and 95% CIs for the linear association between adiposity and all-cause mortality (minimally vs. main/fully adjusted model)

​	N_e_/N_t_	HR^a^ (95% CI) – minimally adjusted model	HR^b^ (95% CI) – main/fully adjusted
BMI (per 5 kg/m^2^)	​	​	​
Pre- or post-diagnosis combined	680/3,760	1.32 (1.20–1.44)	1.30 (1.18–1.44)
Pre-diagnosis	262/1,390	1.16 (0.99–1.36)	1.25 (1.05–1.48)
Post-diagnosis	418/2,370	1.42 (1.27–1.59)	1.37 (1.21–1.54)
Waist circumference (per 10 cm)	​	​	​
Pre- or post-diagnosis combined	680/3,760	1.29 (1.21–1.38)	1.28 (1.19–1.37)
Pre-diagnosis	262/1,390	1.20 (1.07–1.36)	1.27 (1.12–1.44)
Post-diagnosis	418/2,370	1.35 (1.24–1.48)	1.30 (1.19–1.42)
Hip circumference (per 10 cm)	​	​	​
Pre- or post-diagnosis combined	680/3,760	1.34 (1.21–1.49)	1.34 (1.21–1.49)
Pre-diagnosis	262/1,390	1.13 (0.95–1.36)	1.21 (1.01–1.46)
Post-diagnosis	418/2,370	1.46 (1.29–1.66)	1.43 (1.26–1.62)
Waist-to-hip ratio (per 0.1 U)	​	​	​
Pre- or post-diagnosis combined	680/3,760	1.43 (1.27–1.61)	1.35 (1.20–1.53)
Pre-diagnosis	262/1,390	1.42 (1.17–1.73)	1.49 (1.21–1.83)
Post-diagnosis	418/2,370	1.44 (1.24–1.67)	1.30 (1.11–1.52)
Waist-to-height ratio (per 0.1 U)	​	​	​
Pre- or post-diagnosis combined	680/3,760	1.56 (1.39–1.75)	1.51 (1.33–1.70)
Pre-diagnosis	262/1,390	1.39 (1.14–1.69)	1.51 (1.22–1.86)
Post-diagnosis	418/2,370	1.68 (1.45–1.94)	1.56 (1.33–1.82)
Body fat percentage (per 5% body fat increase)^c^	​	​	​
Pre- or post-diagnosis combined	656/3,685	1.27 (1.18–1.36)	1.24 (1.15–1.34)
Pre-diagnosis	253/1,363	1.10 (0.98–1.24)	1.13 (1.00–1.28)
Post-diagnosis	403/2,322	1.38 (1.26–1.51)	1.33 (1.21–1.46)

Abbreviations: Ne, number of events; Nt, total number of men with prostate cancer.

a Model adjusted for age of diagnosis and year of diagnosis – same individuals as in the fully adjusted model.

b Model adjusted for age of diagnosis, year of diagnosis, smoking status (categorical as never, current, or previous), physical activity (continuous as sum of excess MET-hours/week of walking and moderate and vigorous activity), sedentary activities (continuous as the sum of time spent watching TV, using a computer screen, or driving in hours/day), Townsend deprivation index (in quintiles), and alcohol intake frequency (categorical as never, special occasions only, one to three times monthly, once or twice weekly, or daily or almost daily).

^a,b^Models additionally stratified by the UK Biobank center. The date of each respective assessment visit (according to the period that the individual was selected from) was considered as the start of follow-up (entry time). The date of death or censoring (December 31, 2020) was considered as the end of follow-up (exit time). Two individuals with a death date but no death cause were included in all-cause mortality models.

c For the analyses of body fat percentage, the subset of individuals (3,685 of the 3,760) with data for body fat percentage assessed by bioelectrical impedance was utilized.

**Table 3:** Cox proportional HRs and 95% CIs for the linear association between adiposity and prostate cancer–specific mortality (minimally versus main/fully adjusted model)

​	N_e_/N_t_	HR^a^ (95% CI) – minimally adjusted model	HR^b^ (95% CI) – main/fully adjusted
BMI (per 5 kg/m^2^)	​	​	​
Cox model^c^	​	​	​
Pre- or post-diagnosis combined	331/3,760	1.30 (1.14–1.48)	1.33 (1.15–1.52)
Pre-diagnosis	124/1,390	1.09 (0.86–1.38)	1.18 (0.92–1.51)
Post-diagnosis	207/2,370	1.43 (1.21–1.69)	1.43 (1.20–1.69)
Fine–Gray model^d^	​	​	​
Pre- or post-diagnosis combined	331/3,760	1.29 (1.14–1.47)	1.31 (1.16–1.50)
Pre-diagnosis	124/1,390	1.06 (0.83–1.34)	1.14 (0.89–1.47)
Post-diagnosis	207/2,370	1.40 (1.25–1.61)	1.43 (1.24–1.69)
Waist circumference (per 10 cm)	​	​	​
Cox model^c^	​	​	​
Pre- or post-diagnosis combined	331/3,760	1.29 (1.17–1.42)	1.30 (1.18–1.44)
Pre-diagnosis	124/1,390	1.21 (1.02–1.42)	1.30 (1.08–1.55)
Post-diagnosis	207/2,370	1.33 (1.18–1.51)	1.32 (1.16–1.51)
Fine–Gray model^d^	​	​	​
Pre- or post-diagnosis combined	331/3,760	1.28 (1.16–1.35)	1.31 (1.18–1.44)
Pre-diagnosis	124/1,390	1.17 (0.99–1.34)	1.26 (1.06–1.49)
Post-diagnosis	207/2,370	1.35 (1.20–1.48)	1.35 (1.20–1.48)
Hip circumference (per 10 cm)	​	​	​
Cox model^c^	​	​	​
Pre- or post-diagnosis combined	331/3,760	1.39 (1.21–1.61)	1.42 (1.23–1.65)
Pre-diagnosis	124/1,390	1.17 (0.90–1.51)	1.29 (0.98–1.69)
Post-diagnosis	207/2,370	1.51 (1.27–1.79)	1.49 (1.24–1.79)
Fine–Gray model^d^	​	​	​
Pre- or post-diagnosis combined	331/3,760	1.34 (1.21–1.63)	1.41 (1.23–1.63)
Pre-diagnosis	124/1,390	1.15 (0.88–1.48)	1.26 (0.96–1.66)
Post-diagnosis	207/2,370	1.51 (1.29–1.79)	1.51 (1.28–1.79)
Waist-to-hip ratio (per 0.1 U)	​	​	​
Cox model^c^	​	​	​
Pre- or post-diagnosis combined	331/3,760	1.35 (1.14–1.60)	1.34 (1.12–1.60)
Pre-diagnosis	124/1,390	1.41 (1.06–1.89)	1.51 (1.11–2.03)
Post-diagnosis	207/2,370	1.34 (1.08–1.66)	1.30 (1.03–1.63)
Fine-Gray model^d^	​	​	​
Pre- or post-diagnosis combined	331/3,760	1.35 (1.14–1.59)	1.35 (1.14–1.60)
Pre-diagnosis	124/1,390	1.32 (1.01–1.73)	1.42 (1.07–1.88)
Post-diagnosis	207/2,370	1.36 (1.11–1.67)	1.34 (1.07–1.67)
Waist-to-height ratio (per 0.1 U)	​	​	​
Cox model^c^	​	​	​
Pre- or post-diagnosis combined	331/3,760	1.46 (1.24–1.73)	1.48 (1.24–1.77)
Pre-diagnosis	124/1,390	1.25 (0.94–1.67)	1.38 (1.01–1.87)
Post-diagnosis	207/2,370	1.60 (1.30–1.97)	1.58 (1.26–1.98)
Fine–Gray model^d^	​	​	​
Pre- or post-diagnosis combined	331/3,760	1.46 (1.24–1.69)	1.48 (1.25–1.74)
Pre-diagnosis	124/1,390	1.18 (0.89–1.56)	1.29 (0.98–1.72)
Post-diagnosis	207/2,370	1.61 (1.34–1.94)	1.62 (1.33–1.97)
Body fat percentage (per 5% body fat increase)^e^	​	​	​
Cox model^c^	​	​	​
Pre- or post-diagnosis combined	320/3,685	1.25 (1.12–1.38)	1.26 (1.13–1.41)
Pre-diagnosis	119/1,363	1.06 (0.89–1.25)	1.10 (0.92–1.32)
Post-diagnosis	201/2,322	1.36 (1.20–1.55)	1.36 (1.19–1.56)
Fine–Gray model^d^	​	​	​
Pre- or post-diagnosis combined	320/3,685	1.23 (1.10–1.34)	1.25 (1.12–1.40)
Pre-diagnosis	119/1,363	1.05 (0.86–1.28)	1.09 (0.90–1.31)
Post-diagnosis	201/2,322	1.36 (1.20–1.54)	1.37 (1.19–1.54)

The sum of prostate cancer deaths (Table 3) and non–prostate cancer mortality deaths (Table 4) does not add up to the total number of all-cause deaths (in the all-cause mortality analysis, Table 2) as two individuals have a death date but not death cause (according to the ICD-10). These individuals were included only in the analyses of all-cause mortality.

Abbreviations: N_e,_ number of events; N_t,_ total number of men with prostate cancer.

a Model adjusted for age of diagnosis and year of diagnosis – same individuals as in the fully adjusted model.

b Model adjusted for age of diagnosis, year of diagnosis, smoking status (categorical as never, current, or previous), physical activity (continuous as sum of excess MET-hours/week of walking and moderate and vigorous activity), sedentary activities (continuous as sum of time spent watching TV, using a computer screen, or driving in hours/day), Townsend deprivation index (in quintiles), and alcohol intake frequency (categorical as never, special occasions only, one to three times monthly, once or twice weekly, or daily or almost daily).

^a,b^Models additionally stratified by the UK Biobank center. The date of each respective assessment visit (according to the period that the individual was selected from) was considered as the start of follow-up (entry time). The date of death or censoring (December 31, 2020) was considered as the end of follow-up (exit time).

c Cox model treating non–prostate cancer deaths as censored.

d Fine–Gray sub-distribution hazard model; competing risk is death due to other causes and not specific to prostate cancer.

e For the analyses of body fat percentage, the subset of individuals (3,685 of the 3,760) with data for body fat percentage assessed by bioelectrical impedance was utilized.

**Table 4:** Cox proportional HRs and 95% CIs for the linear association between adiposity and non–prostate cancer mortality (minimally vs. main/fully adjusted model)

​	N_e_/N_t_	HR^a^ (95% CI) – minimally adjusted model	HR^b^ (95% CI) – main/fully adjusted
BMI (per 5 kg/m^2^)	​	​	​
Cox model^c^	​	​	​
Pre- or post-diagnosis combined	347/3,760	1.34 (1.17–1.52)	1.28 (1.12–1.47)
Pre-diagnosis	137/1,390	1.22 (0.98–1.52)	1.29 (1.02–1.63)
Post-diagnosis	210/2,370	1.42 (1.20–1.67)	1.31 (1.11–1.56)
Fine–Gray model^d^	​	​	​
Pre- or post-diagnosis combined	347/3,760	1.31 (1.15–1.50)	1.26 (1.09–1.46)
Pre-diagnosis	137/1,390	1.20 (0.96–1.52)	1.26 (0.98–1.63)
Post-diagnosis	210/2,370	1.40 (1.22–1.65)	1.29 (1.08–1.55)
Waist circumference (per 10 cm)	​	​	​
Cox model^c^	​	​	​
Pre- or post-diagnosis combined	347/3,760	1.30 (1.18–1.43)	1.25 (1.13–1.38)
Pre-diagnosis	137/1,390	1.20 (1.02–1.41)	1.23 (1.04–1.47)
Post-diagnosis	210/2,370	1.38 (1.22–1.55)	1.28 (1.12–1.45)
Fine–Gray model^d^	​	​	​
Pre- or post-diagnosis combined	347/3,760	1.27 (1.15–1.40)	1.21 (1.08–1.36)
Pre-diagnosis	137/1,390	1.16 (0.98–1.38)	1.18 (0.97–1.44)
Post-diagnosis	210/2,370	1.35 (1.20–1.51)	1.26 (1.09–1.42)
Hip circumference (per 10 cm)	​	​	​
Cox model^c^	​	​	​
Pre- or post-diagnosis combined	347/3,760	1.29 (1.12–1.49)	1.26 (1.09–1.46)
Pre-diagnosis	137/1,390	1.09 (0.85–1.40)	1.13 (0.87–1.46)
Post-diagnosis	210/2,370	1.42 (1.19–1.70)	1.37 (1.14–1.64)
Fine–Gray model^d^	​	​	​
Pre- or post-diagnosis combined	347/3,760	1.24 (1.06–1.45)	1.21 (1.03–1.41)
Pre-diagnosis	137/1,390	1.06 (0.82–1.35)	1.08 (0.83–1.42)
Post-diagnosis	210/2,370	1.37 (1.14–1.63)	1.32 (1.09–1.58)
Waist-to-hip ratio (per 0.1 U)	​	​	​
Cox model^c^	​	​	​
Pre- or post-diagnosis combined	347/3,760	1.51 (1.28–1.78)	1.36 (1.15–1.62)
Pre-diagnosis	137/1,390	1.43 (1.09–1.87)	1.45 (1.10–1.93)
Post-diagnosis	210/2,370	1.56 (1.27–1.93)	1.31 (1.05–1.62)
Fine–Gray model^d^	​	​	​
Pre- or post-diagnosis combined	347/3,760	1.48 (1.24–1.76)	1.33 (1.11–1.59)
Pre-diagnosis	137/1,390	1.39 (1.02–1.89)	1.40 (1.01–1.96)
Post-diagnosis	210/2,370	1.54 (1.25–1.90)	1.30 (1.05–1.60)
Waist-to-height ratio (per 0.1 U)	​	​	​
Cox model^c^	​	​	​
Pre- or post-diagnosis combined	347/3,760	1.65 (1.41–1.94)	1.54 (1.30–1.82)
Pre-diagnosis	137/1,390	1.51 (1.15–1.97)	1.61 (1.20–2.16)
Post-diagnosis	210/2,370	1.77 (1.44–2.17)	1.54 (1.24–1.91)
Fine–Gray model^d^	​	​	​
Pre- or post-diagnosis combined	347/3,760	1.58 (1.34–1.87)	1.46 (1.22–1.76)
Pre-diagnosis	137/1,390	1.44 (1.08–1.93)	1.51 (1.09–2.09)
Post-diagnosis	210/2,370	1.71 (1.39–2.09)	1.49 (1.19–1.86)
Body fat percentage (per 5% body fat increase)^e^	​	​	​
Cox model^c^	​	​	​
Pre- or post-diagnosis combined	334/3,685	1.29 (1.16–1.42)	1.22 (1.10–1.36)
Pre-diagnosis	133/1,363	1.15 (0.97–1.35)	1.14 (0.96–1.36)
Post-diagnosis	201/2,322	1.40 (1.22–1.59)	1.30 (1.13–1.49)
Fine–Gray model^d^	​	​	​
Pre- or post-diagnosis combined	334/3,685	1.26 (1.14–1.41)	1.20 (1.08–1.34)
Pre-diagnosis	133/1,363	1.14 (0.96–1.37)	1.13 (0.94–1.38)
Post-diagnosis	201/2,322	1.38 (1.22–1.57)	1.28 (1.12–1.47)

The sum of prostate cancer deaths (Table 3) and non–prostate cancer mortality deaths (Table 4) does not add up to the total number of all-cause deaths (in the all-cause mortality analysis, Table 2) as two individuals have a death date but not death cause (according to the ICD-10). These individuals were included only in the analyses of all-cause mortality.

Abbreviations: Ne, number of events; Nt, total number of men with prostate cancer.

a Model adjusted for age of diagnosis and year of diagnosis – same individuals as in the fully adjusted model.

b Model adjusted for age of diagnosis, year of diagnosis, smoking status (categorical as never, current, or previous), physical activity (continuous as sum of excess MET-hours/week of walking and moderate and vigorous activity), sedentary activities (continuous as sum of time spent watching TV, using a computer screen, or driving in hours/day), Townsend deprivation index (in quintiles), and alcohol intake frequency (categorical as never, special occasions only, one to three times monthly, once or twice weekly, or daily or almost daily).

^a,b^Models additionally stratified by the UK Biobank center. The date of each respective assessment visit (according to the period that the individual was selected from) was considered as the start of follow-up (entry time). The date of death or censoring (December 31, 2020) was considered as the end of follow-up (exit time).

c Cox model is treating prostate cancer–specific deaths as censored.

d Fine–Gray sub-distribution hazard model; competing risk is prostate cancer–specific mortality (death due to prostate cancer).

e For the analyses of body fat percentage, the subset of individuals (3,685 of the 3,760) with data for body fat percentage assessed by bioelectrical impedance was utilized.

#### Waist circumference

Analysis of waist circumference assessed before or after diagnosis combined showed a linear increase in the rate of all-cause (HR per 10 cm = 1.28; 95% CI, 1.19–1.37), prostate cancer–specific (HR per 10 cm = 1.30; 95% CI, 1.18–1.44), and non–prostate cancer mortality (HR per 10 cm = 1.25; 95% CI, 1.13–1.38). Similar positive associations were observed for pre-diagnosis waist circumference (HR per 10 cm = 1.27; 95% CI, 1.12–1.44 for all cause, HR per 10 cm = 1.30; 95% CI, 1.08–1.55 for prostate cancer–specific, and HR per 10 cm = 1.23; 95% CI, 1.04–1.47 for non–prostate cancer mortality) and for post-diagnosis waist circumference (HR per 10 cm = 1.30; 95% CI, 1.19–1.42 for all-cause, HR per 10 cm = 1.32; 95% CI, 1.16–1.51 for prostate cancer–specific, and HR per 10 cm = 1.28; 95% CI, 1.12–1.45 for non–prostate cancer mortality) as shown in Tables 2–4 and Supplementary Fig. S2.

#### Hip circumference

Hip circumference assessed before or after diagnosis combined was associated with an increase in the rate of all-cause (HR per 10 cm = 1.34; 95% CI, 1.21–1.49), prostate cancer–specific (HR per 10 cm = 1.42; 95% CI, 1.23–1.65), and non–prostate cancer mortality (HR per 10 cm = 1.26; 95% CI, 1.09–1.46) that was similar for pre- (HR per 10 cm = 1.21; 95% CI, 1.01–1.46 for all-cause, HR per 10 cm = 1.29; 95% CI, 0.98–1.69 for prostate cancer–specific, and HR per 10 cm = 1.13; 95% CI, 0.87–1.46 for non–prostate cancer mortality) and for post-diagnosis hip circumference (HR per 10 cm = 1.43; 95% CI, 1.26–1.62 for all-cause, HR per 10 cm = 1.49; 95% CI, 1.24–1.79 for prostate cancer–specific mortality, and HR per 10 cm = 1.37; 95% CI, 1.14–1.64 for non–prostate cancer mortality) as shown in Tables 2–4 and Supplementary Fig. S3.

#### Waist-to-hip ratio

A positive association was also observed between waist-to-hip ratio assessed before or after diagnosis combined and all-cause (HR per 0.1 U = 1.35; 95% CI, 1.20–1.53), prostate cancer–specific (HR per 0.1 U = 1.34; 95% CI, 1.12–1.60), and non–prostate cancer mortality (HR per 0.1 U = 1.36; 95% CI, 1.15–1.62) that was similar for pre- (HR per 0.1 U = 1.49; 95% CI, 1.21–1.83 for all-cause, HR per 0.1 U = 1.51; 95% CI, 1.11–2.03 for prostate cancer–specific mortality, and HR per 0.1 U = 1.45; 95% CI, 1.10–1.93 for non–prostate cancer mortality) and post-diagnosis waist-to-hip ratio (HR per 0.1 U = 1.30; 95% CI, 1.11–1.52 for all-cause, HR per 0.1 U = 1.30; 95% CI, 1.03–1.63 for prostate cancer–specific, and HR per 0.1 U = 1.31; 95% CI, 1.05–1.62 for non–prostate cancer mortality) as shown in Tables 2–4 and Supplementary Fig. S4.

#### Waist-to-height ratio

Waist-to-height ratio assessed before or after diagnosis combined was associated with a higher rate of all-cause (HR per 0.1 U = 1.51; 95% CI, 1.33–1.70), prostate cancer–specific (HR per 0.1 U = 1.48; 95% CI, 1.24–1.77), and non–prostate cancer mortality (HR per 0.1 U = 1.54; 95% CI, 1.30–1.82). Similar positive associations were seen for pre- (HR per 0.1 U = 1.51; 95% CI, 1.22–1.86 for all-cause, HR per 0.1 U = 1.38; 95% CI, 1.01–1.87 for prostate cancer–specific, and HR per 0.1 U = 1.61; 95% CI, 1.20–2.16 for non–prostate cancer mortality) and post-diagnosis waist-to-height ratio (HR per 0.1 U = 1.56; 95% CI, 1.33–1.82 for all-cause, HR per 0.1 U = 1.58; 95% CI, 1.26–1.98 for prostate cancer–specific, and HR per 0.1 U = 1.54; 95% CI, 1.24–1.91 for non–prostate cancer mortality) as shown in Tables 2–4 and Supplementary Fig. S5.

#### Body fat percentage assessed by bioelectrical impedance

Of the 3,760 men who represented the analytic sample of the present study, 3,685 (98%) had body fat percentage data measured by bioelectrical impedance. A positive association was observed between body fat percentage assessed close to diagnosis (before and after diagnosis combined) and all-cause (HR per 5% increase in total body fat percentage = 1.24; 95% CI, 1.15–1.34), prostate cancer–specific (HR per 5% increase in total body fat percentage = 1.26; 95% CI, 1.13–1.41), and non–prostate cancer (HR per 5% increase in body fat percentage = 1.22; 95% CI, 1.10–1.36) mortality. Positive associations were also observed in the post-diagnosis analysis for all-cause (HR per 5% increase in total body fat percentage = 1.33; 95% CI, 1.21–1.46), prostate cancer–specific (HR per 5% increase in total body fat percentage = 1.36; 95% CI, 1.19–1.56), and non–prostate cancer (HR = 1.30; 95% CI, 1.13–1.49) mortality. For pre-diagnosis body fat percentage, associations in the positive direction were observed for all-cause (HR per 5% increase in total body fat percentage = 1.13; 95% CI, 1.00–1.28), prostate cancer–specific (HR per 5% increase in total body fat percentage = 1.10; 95% CI, 0.92–1.32), and non–prostate cancer (HR per 5% increase in total body fat percentage = 1.14; 95% CI, 0.96–1.36) mortality as shown in Tables 2–4 and Supplementary Fig. S6.

#### Nonlinear associations

The nonlinear analyses indicated clear positive associations for overall and post-diagnosis adiposity and mortality across all the adiposity indices (BMI, waist circumference, hip circumference, waist-to-hip ratio, waist-to-height ratio, and body fat percentage). For pre-diagnosis adiposity, the positive association with mortality outcomes was more evident above the median value of each adiposity index (Fig. 2; Supplementary Figs. S2–S6).

#### Subgroup and sensitivity analyses

Positive associations of similar magnitude were observed across all the adiposity indices when comparing the fully adjusted model (main model) with the minimally adjusted model (for age and year of diagnosis). Accounting for the potential influence of competing causes of death in the prostate cancer–specific mortality and non–prostate cancer mortality models did not alter the associations (Tables 3 and 4).

The analysis by each year of adiposity measurement (i.e., in each year before and after diagnosis) yielded similar results to the main analyses for all indices. In some of the analyses, mainly for prostate cancer–specific mortality, the 95% CIs crossed the null value (Supplementary Table S5).

Of the total men with adiposity data close to diagnosis, 56% had prevalent prostate cancer. Similar positive associations were observed in analyses including men who had only prevalent versus only incident prostate cancer (Supplementary Table S6).

When comparing important variables for potential selection bias, no material differences were observed in participants included and excluded from our study sample (Supplementary Table S7). Analysis stratified by smoking status showed little differences. There was evidence of positive associations in never and previous smokers who represented the majority (∼92%) of the study population, consistent with the main analysis and null associations among current smokers (Supplementary Table S8).

Similar positive associations to the main analyses were also observed in the analyses after exclusion of the first year, and in separate analysis, the first 2 years of follow-up (Supplementary Table S9).

## Discussion

The present study included men with prostate cancer from the UK Biobank who had adiposity data measured close to diagnosis. Overall, positive associations were observed in the analysis of BMI with all-cause, prostate cancer–specific, and non–prostate cancer mortality. Analyses of waist circumference, hip circumference, waist-to-hip ratio, waist-to-height ratio, and body fat percentage (assessed by bioelectrical impedance) yielded positive associations of similar magnitude as for BMI. Results of the subgroup and sensitivity analyses indicated generally consistent positive associations.

Evidence on the associations between adiposity assessed close to or after diagnosis and mortality in men with prostate cancer is emerging but still inconclusive (8, 12). Observational studies so far have reported mixed results; some reported positive associations (11, 52) and some inverse associations (53–55), whereas others found no associations with mortality (56–59). Few studies to date investigated other adiposity indices such as waist circumference (56, 57, 60) that could better predict adiposity in men as compared with BMI as adipose tissue is usually accumulated centrally in the abdomen (61, 62).

Most meta-analyses in patients with prostate cancer to date (published between 2011 and 2021) reported null associations between at/post-diagnosis BMI and prostate cancer–specific or all-cause mortality (63–65). One of the published meta-analyses (65) identified a small increase in the rate of all-cause mortality. Our meta-analysis, published in 2023, explored linear and nonlinear associations and identified a J-shaped association between adiposity assessed at/after diagnosis and all-cause and prostate cancer–specific mortality (12), with a clear positive and linear association for BMI higher than 28 kg/m^2^.

The present study provided a large sample size for all the adiposity indices after removing a small number of individuals with missing data in covariates (*N* = 3,760 in the largest analysis of pre- or post-diagnosis adiposity combined). It represents the largest analysis of pre- or post-diagnosis adiposity combined, and the total number of deaths (*N* = 680, all-cause; *N* = 331, prostate cancer–specific deaths) was higher than the average number of deaths included in previous observational studies on prostate cancer survival (*N* = 550, all-cause, *N* = 217 prostate cancer–specific deaths).

Findings of the present analyses indicated that elevated adiposity is associated with worse survival outcomes. The relationship between obesity and outcomes after cancer diagnosis is biologically complex and (66) the biological mechanisms that link obesity to worse survival outcomes have not yet been clarified (67, 68). Plausible explanations include obesity-induced inflammatory and metabolic changes in the adipose tissue microenvironment that could be drivers of tumor progression to metastasis, could affect response to treatments (e.g., resistance to androgen suppression therapies), and lead to a higher risk of death (69–71). Moreover, activated macrophages in the adipose tissue of individuals with obesity could enhance the transcription of pro-inflammatory genes in prostate tumor cells (70, 72, 73). Apart from the direct consequences of adiposity, men with prostate cancer and obesity could possibly experience poorer treatment-related outcomes or worse side effects (62, 68).

It is possible for individuals with obesity to require alternative treatment options or to receive suboptimal treatment and this is often under-reported in clinical studies (74). Individuals with obesity, as compared with individuals with normal weight, could have delayed diagnosis or under-staged tumor at diagnosis resulting from less accurate biopsy tests or enlarged prostates and potentially lower PSA levels (67, 75, 76). It is possible that hormone therapy could lead to metabolic and body composition changes including increase in fat mass and consequently obesity if it remains uncontrolled. The interaction between body composition and various treatment modalities with respect to long-term survival outcomes needs to be better understood (67).

Targeted non-pharmacologic approaches, including appropriate lifestyle strategies, could be designed and integrated into routine care of patients with cancer aiming to improve prognosis (70). Existing evidence supports that diet-induced weight loss and exercise interventions could prevent adverse effects of obesity on lymphatic function (77, 78). Randomized controlled trials in patients with cancer that investigate “hard” primary outcomes including mortality are limited and challenging to conduct but more timely now as the evidence from observational and basic science studies is emerging (79). Carefully designed observational studies and randomized controlled trials with surrogate disease endpoints and biomarkers are essential to indicate whether certain health behaviors could be beneficial or not (6, 79, 80). Existing evidence suggests that a balanced, carefully designed diet (81) and/or other appropriate lifestyle modifications (73) could affect inflammatory markers within the tumor microenvironment and potentially improve clinical outcomes (e.g., better treatment response and survival; ref. 81). Genomic testing can also help identify tumor subtypes that might benefit from additional interventions and molecular subtypes that would not (68). It will also be important to find cost-efficient ways to identify individuals with excess body fat who might be in the “normal-range” BMI category (70).

Strengths of this work include the detailed prospective data on measured adiposity indices close to prostate cancer diagnosis. The relatively large sample size of men with adiposity data close to diagnosis allowed us to explore the associations between adiposity indices that have been less well investigated in observational studies to date i.e., waist and hip circumference, waist-to-hip ratio, waist-to-height ratio, and body composition assessed by bioelectrical impedance (body fat percentage) in addition to BMI. There are limitations of body fat assessed by bioelectrical impedance, including that it often underestimates fat-free mass in individuals with normal weight and it might overestimate it in individuals with obesity (82). However, a previous study in the UK Biobank showed strong correlation between bioelectrical impedance–derived and dual-energy absorptiometry–derived body composition measures (83). Very few patients with prostate cancer from our analytic sample had total body fat percentage measured by dual-energy X-ray absorptiometry (51), which is often considered a more accurate measurement of body composition (83); therefore, an analysis was not feasible. Important sensitivity analyses were performed to explore potential reverse causation and selection bias as well as the influence of competing causes of death on prostate cancer–specific mortality. Results indicated that the probability of such biases is likely to be small.

Undetected disease progression or tumor recurrence could bias the associations in studies of mortality outcomes (84); however, we did not have such data available. Instead, we performed analysis separately for each year of adiposity measurement before diagnosis (for the first year before diagnosis and separately for the second year before diagnosis) and at each year after diagnosis (up to 5 years after diagnosis). This analysis could provide additional information about the associations at different phases of the cancer survivorship that might be affected differently by reverse causation. Results according to each year of adiposity measurement were generally similar. In addition, removing the first year of follow-up and then in a separate analysis removing the second year of follow-up after the adiposity assessment also yielded similar results to the main analyses.

Lack of data on cancer treatments, stage, and grade did not allow us to account for these variables in the analyses although they are well-known predictors of outcomes after prostate cancer diagnosis. It also hindered investigation of whether the associations differ according to tumor characteristics. Plans are underway to expand the linkage of the UK Biobank database to national datasets and include information on tumor aggressiveness (stage and grade), tumor morphology, and treatment (radiotherapy, chemotherapy, immunotherapy, and hormone treatments; ref. 25). These variables, however, were not available at the time of the present analysis (24, 85, 86). Adjusting by the year of cancer diagnosis could mitigate this limitation to a small degree as it reflects developments in prostate cancer detection and treatments across time (87). Results of the present study in the UK Biobank showed positive associations without stage and grade adjustment similar to the positive associations that were seen in EPIC (20), in which it was possible to adjust for stage and grade. Other studies that investigated the associations between BMI and mortality outcomes in patients with prostate cancer reported that models additionally adjusted for variables including treatment, prostate cancer risk category, tumor–node–metastasis stage, and grade (52, 58, 88, 89) did not materially alter results.

Another limitation of the present study is that the UK Biobank cohort comprised mainly White men; therefore, the observed associations may not be generalizable to other populations. Additional studies are needed in less well-investigated ethnic groups to explore whether there are any differences in the body composition–mortality association in more diverse subgroups of race/ethnicity (90).

It is important to investigate the timing of obesity in relation to outcomes after prostate cancer diagnosis as well as the cumulative effect of obesity in terms of potential changes in weight (11, 68). The present study as well as most observational studies to date utilized a single timepoint adiposity measure instead of multiple longitudinal measurements. Repeated adiposity data (i.e., measured in more than one adiposity assessment according to our specific eligibility criteria) were limited. Few men (<1%) had post-diagnosis data measured at more than one assessment visit (e.g., at baseline and again at re-survey or at the re-survey and again at the first imaging visit), which is a limitation of the present study that we could not perform such an analysis. The pre- or post-diagnosis adiposity combined analysis was the best possible way to capture adiposity in the period close to the diagnosis. When possible, future studies should perform time-varying analyses.

## Conclusion

The present study provided evidence that obesity in men with prostate cancer could be associated with higher mortality rates. It is important to acquire more evidence on the associations between lifestyle factors, such as adiposity, and prostate cancer prognosis from carefully designed observational and interventional studies. The precise mechanisms behind the potential associations need to be clarified further in future studies to provide evidence-based lifestyle advice that will have the potential to reduce mortality and improve long-term prostate cancer outcomes.

## Supplementary Material

Supplementary Figure 1 Directed acyclic graph showing potential causal relations between the variables considered in the present study.

Supplementary Figure 2 Restricted cubic spline analysis for waist circumference assessed close to diagnosis and all-cause, prostate cancer-specific and non-prostate cancer mortality.

Supplementary Figure 3 Restricted cubic spline analysis for hip circumference assessed close to diagnosis and all-cause, prostate cancer-specific and non-prostate cancer mortality.

Supplementary Figure 4 Restricted cubic spline analysis for waist-to-hip ratio assessed close to diagnosis and all-cause, prostate cancer-specific and non-prostate cancer mortality.

Supplementary Figure 5 Restricted cubic spline analysis for waist-to-height ratio assessed close to diagnosis and all-cause, prostate cancer-specific and non-prostate cancer mortality.

Supplementary Figure 6 Restricted cubic spline analysis for body fat percentage (%) assessed close to diagnosis and all-cause, prostate cancer-specific and non-prostate cancer mortality.

Supplementary Table 1 Relevant covariates available in UK Biobank database based on subject matter knowledge.

Supplementary Table 2 Missing covariate data from the 3,916 men (prior to elimination of individuals with missing data).

Supplementary Table 3 Important demographic characteristics of the 3,760 men with prostate cancer in UK Biobank according to the BMI, WHO categories.

Supplementary Table 4 Categorical analysis according to the BMI WHO categories and mortality.

Supplementary Table 5 Sensitivity analysis, in each year of adiposity measurement. Cox Proportional HRs and 95%CIs for the linear association between adiposity by each year of adiposity measurement and all-cause and prostate cancer-specific mortality.

Supplementary Table 6 Cox Proportional Hazard Ratios and 95%CIs for the linear association between adiposity close to diagnosis (pre- or post-diagnosis adiposity combined) and all-cause and prostate cancer-specific mortality in individuals with incident versus prevalent prostate cancer (fully adjusted/main model).

Supplementary Table 7 Comparison of major lifestyle characteristics of men with BMI data according to the eligibility criteria of the present study versus those excluded because they did not have adiposity data and versus those included in the main models.

Supplementary Table 8 Sub-group analysis by smoking for men with BMI close to prostate cancer diagnosis (pre- or post-diagnosis BMI combined).

Supplementary Table 9 Cox Proportional HRs and 95%CIs for the linear association between adiposity after removing 1) the first year of follow-up and 2) the first two years of follow-up in relation to all-cause and prostate cancer-specific mortality (main/fully adjusted model).

## References

[1] Sung H , Ferlay J, Siegel RL, Laversanne M, Soerjomataram I, Jemal A, . Global cancer statistics 2020: GLOBOCAN estimates of incidence and mortality worldwide for 36 cancers in 185 countries. CA Cancer J Clin 2021;71:209–49. 33538338 10.3322/caac.21660

[2] Global cancer observatory - data version: Globocan 2022 - 08.02.2024. [Cited 2024 May]. Available from: https://gco.iarc.fr/en.

[3] Cancer Research UK. [Cited 2020 Dec 20]. Available from: https://www.cancerresearchuk.org/health-professional/cancer-statistics/statistics-by-cancer-type/prostate-cancer/incidence#heading-Three.

[4] Rebello RJ , Oing C, Knudsen KE, Loeb S, Johnson DC, Reiter RE, . Prostate cancer. Nat Rev Dis Primers 2021;7:9. 33542230 10.1038/s41572-020-00243-0

[5] Barsouk A , Padala SA, Vakiti A, Mohammed A, Saginala K, Thandra KC, . Epidemiology, staging and management of prostate cancer. Med Sci (Basel) 2020;8:28. 32698438 10.3390/medsci8030028 PMC7565452

[6] Spei ME , Bellos I, Samoli E, Benetou V. Post-diagnosis dietary patterns among cancer survivors in relation to all-cause mortality and cancer-specific mortality: a systematic review and meta-analysis of cohort studies. Nutrients 2023;15:3860. 37686892 10.3390/nu15173860 PMC10490392

[7] Lagergren P , Schandl A, Aaronson NK, Adami HO, de Lorenzo F, Denis L, . Cancer survivorship: an integral part of Europe’s research agenda. Mol Oncol 2019;13:624–35. 30552794 10.1002/1878-0261.12428 PMC6396379

[8] Rock CL , Thomson CA, Sullivan KR, Howe CL, Kushi LH, Caan BJ, . American Cancer Society nutrition and physical activity guideline for cancer survivors. CA Cancer J Clin 2022;72:230–62. 35294043 10.3322/caac.21719

[9] World Cancer Research Fund International/American Institute for Cancer Research . Diet, nutrition, physical activity and cancer: a global perspective. Continuous update project report. 2018 [cited 2024 Jun]. Available from: https://www.wcrf.org/dietandcancer.

[10] Bergengren O , Pekala KR, Matsoukas K, Fainberg J, Mungovan SF, Bratt O, . 2022 update on prostate cancer Epidemiology and risk factors-A systematic review. Eur Urol 2023;84:191–206. 37202314 10.1016/j.eururo.2023.04.021 PMC10851915

[11] Troeschel AN , Hartman TJ, Jacobs EJ, Stevens VL, Gansler T, Flanders WD, . Postdiagnosis body mass index, weight change, and mortality from prostate cancer, cardiovascular disease, and all causes among survivors of nonmetastatic prostate cancer. J Clin Oncol 2020;38:2018–27. 32250715 10.1200/JCO.19.02185 PMC8265380

[12] Cariolou M , Markozannes G, Becerra-Tomás N, Vieira R, Balducci K, Aune D, . Association between adiposity after diagnosis of prostate cancer and mortality: systematic review and meta-analysis. BMJ Med 2023;2:e000339. 10.1136/bmjmed-2022-000339 PMC10568122 37841967

[13] Perez-Cornago A , Appleby PN, Pischon T, Tsilidis KK, Tjønneland A, Olsen A, . Tall height and obesity are associated with an increased risk of aggressive prostate cancer: results from the EPIC cohort study. BMC Med 2017;15:115. 28701188 10.1186/s12916-017-0876-7 PMC5508687

[14] Kazmi N , Haycock P, Tsilidis K, Lynch BM, Truong T; Practical Consortium CRUK BPC3 CAPS PEGASUS, Martin RM, . Appraising causal relationships of dietary, nutritional and physical-activity exposures with overall and aggressive prostate cancer: two-sample mendelian-randomization study based on 79 148 prostate-cancer cases and 61106 controls. Int J Epidemiol 2020;49:587–96. 31802111 10.1093/ije/dyz235

[15] Discacciati A , Orsini N, Wolk A. Body mass index and incidence of localized and advanced prostate cancer–a dose-response meta-analysis of prospective studies. Ann Oncol 2012;23:1665–71. 22228452 10.1093/annonc/mdr603

[16] Pischon T , Boeing H, Hoffmann K, Bergmann M, Schulze MB, Overvad K, . General and abdominal adiposity and risk of death in Europe. N Engl J Med 2008;359:2105–20. 19005195 10.1056/NEJMoa0801891

[17] World Cancer Research Fund International/American Institute for Cancer Research Continuous Update Project Report . Diet, nutrition, physical activity, and prostate cancer. 2018. [cited 2024 Jun]. Available from: https://www.wcrf.org/wp-content/uploads/2024/10/prostate-cancer-report.pdf.

[18] Pischon T , Boeing H, Weikert S, Allen N, Key T, Johnsen NF, . Body size and risk of prostate cancer in the European prospective investigation into cancer and nutrition. Cancer Epidemiol Biomarkers Prev 2008;17:3252–61. 18990768 10.1158/1055-9965.EPI-08-0609

[19] Perez-Cornago A , Dunneram Y, Watts EL, Key TJ, Travis RC. Adiposity and risk of prostate cancer death: a prospective analysis in UK Biobank and meta-analysis of published studies. BMC Med 2022;20:143. 35509091 10.1186/s12916-022-02336-x PMC9069769

[20] Cariolou M , Christakoudi S, Gunter MJ, Key T, Pérez-Cornago A, Travis R, . Adiposity assessed close to diagnosis and prostate cancer prognosis in the EPIC study. JNCI Cancer Spectr 2024;8:pkae070. 39180334 10.1093/jncics/pkae070 PMC11410200

[21] UK Biobank. [cited 2024 Jun]. Available from: https://www.ukbiobank.ac.uk/.

[22] Collins R . What makes UK Biobank special? Lancet 2012;379:1173–4. 22463865 10.1016/S0140-6736(12)60404-8

[23] Bycroft C , Freeman C, Petkova D, Band G, Elliott LT, Sharp K, . The UK Biobank resource with deep phenotyping and genomic data. Nature 2018;562:203–9. 30305743 10.1038/s41586-018-0579-z PMC6786975

[24] Sudlow C , Gallacher J, Allen N, Beral V, Burton P, Danesh J, . UK biobank: an open access resource for identifying the causes of a wide range of complex diseases of middle and old age. PLoS Med 2015;12:e1001779. 25826379 10.1371/journal.pmed.1001779 PMC4380465

[25] Conroy MC , Lacey B, Bešević J, Omiyale W, Feng Q, Effingham M, . UK Biobank: a globally important resource for cancer research. Br J Cancer 2023;128:519–27. 36402876 10.1038/s41416-022-02053-5 PMC9938115

[26] Trock BJ , Han M, Freedland SJ, Humphreys EB, DeWeese TL, Partin AW, . Prostate cancer-specific survival following salvage radiotherapy vs observation in men with biochemical recurrence after radical prostatectomy. JAMA 2008;299:2760–9. 18560003 10.1001/jama.299.23.2760 PMC3076799

[27] Limonta P , Moretti R, Marzagalli M, Marelli M. Castration Resistant Prostate Cancer: from emerging molecular pathways to targeted therapeutic approaches. Clin Cancer Drugs 2014;1:11–27.

[28] Chetta P , Zadra G. Metabolic reprogramming as an emerging mechanism of resistance to endocrine therapies in prostate cancer. Cancer Drug Resist 2021;4:143–62. 35582011 10.20517/cdr.2020.54 PMC9019185

[29] Rawla P . Epidemiology of prostate cancer. World J Oncol 2019;10:63–89. 31068988 10.14740/wjon1191 PMC6497009

[30] UK Biobank Coordinating Centre; UK Biobank: Protocol for a large-scale prospective epidemiological resource. Protocol No: UKBB-PROT-09–06 (Main Phase); 21 March 2007 (AMENDMENT ONE FINAL). [Cited 2023 Aug 8]. Available from: https://www.ukbiobank.ac.uk/media/gnkeyh2q/study-rationale.pdf.

[31] Hughes RA , Heron J, Sterne JAC, Tilling K. Accounting for missing data in statistical analyses: multiple imputation is not always the answer. Int J Epidemiol 2019;48:1294–304. 30879056 10.1093/ije/dyz032 PMC6693809

[32] Craig CL , Marshall AL, Sjöström M, Bauman AE, Booth ML, Ainsworth BE, . International physical activity questionnaire: 12-country reliability and validity. Med Sci Sports Exerc 2003;35:1381–95. 12900694 10.1249/01.MSS.0000078924.61453.FB

[33] Ainsworth BE , Haskell WL, Herrmann SD, Meckes N, Bassett DR Jr, Tudor-Locke C. . Compendium of physical activities: a second update of codes and MET values. Med Sci Sports Exerc. 2011;43:1575–81. 21681120 10.1249/MSS.0b013e31821ece12

[34] Guo W , Bradbury KE, Reeves GK, Key TJ. Physical activity in relation to body size and composition in women in UK Biobank. Ann Epidemiol 2015;25:406–13.e6. 25749558 10.1016/j.annepidem.2015.01.015

[35] Howley ET . Type of activity: resistance, aerobic and leisure versus occupational physical activity. Med Sci Sports Exerc 2001;33(6 Suppl):S364–9. 11427761 10.1097/00005768-200106001-00005

[36] Armstrong ME , Cairns BJ, Green J, Reeves GK, Beral V; Million Women Study Collaborators. Reported frequency of physical activity in a large epidemiological study: relationship to specific activities and repeatability over time. BMC Med Res Methodol 2011;11:97. 21831330 10.1186/1471-2288-11-97 PMC3145605

[37] Bradbury KE , Guo W, Cairns BJ, Armstrong ME, Key TJ. Association between physical activity and body fat percentage, with adjustment for BMI: a large cross-sectional analysis of UK Biobank. BMJ Open 2017;7:e011843. 10.1136/bmjopen-2016-011843 PMC5372047 28341684

[38] Inan-Eroglu E , Powell L, Hamer M, O'Donovan G, Duncan MJ, Stamatakis E. Is there a link between different types of alcoholic drinks and obesity? An analysis of 280,183 UK Biobank participants. Int J Environ Res Public Health 2020;17:5178. 32709071 10.3390/ijerph17145178 PMC7400254

[39] Drozd M , Pujades-Rodriguez M, Lillie PJ, Straw S, Morgan AW, Kearney MT, . Non-communicable disease, sociodemographic factors, and risk of death from infection: a UK Biobank observational cohort study. Lancet Infect Dis 2021;21:1184–91. 33662324 10.1016/S1473-3099(20)30978-6 PMC8323124

[40] Townsend P , Phillimore P, Beattie A. Health and deprivation: inequality and the North. London (United Kingdom): Taylor & Francis; 2023.

[41] UK Biobank touch-screen questionnaire: final version. [cited 2024 Jun]. Available from: https://biobank.ndph.ox.ac.uk/ukb/ukb/docs/TouchscreenQuestionsMainFinal.pdf.

[42] Christakoudi S , Tsilidis KK, Evangelou E, Riboli E. A Body Shape Index (ABSI), hip index, and risk of cancer in the UK Biobank cohort. Cancer Med 2021;10:5614–28. 34196490 10.1002/cam4.4097 PMC8366087

[43] Fine JP , Gray RJ. A proportional hazards model for the subdistribution of a competing risk. J Am Stat Assoc 1999;94:496–509.

[44] Austin PC , Lee DS, Fine JP. Introduction to the analysis of survival data in the presence of competing risks. Circulation 2016;133:601–9. 26858290 10.1161/CIRCULATIONAHA.115.017719 PMC4741409

[45] Harrell FE Jr . General aspects of fitting regression models. In: Regression modeling strategies: with applications to linear models, logistic and ordinal regression, and survival analysis. Cham (Switzerland): Springer International Publishing; 2015. pp. 13–44.

[46] Schoenfeld D . Partial residuals for the proportional hazards regression model. Biometrika 1982;69:239–41.

[47] Halabi S , Kelly WK, Ma H, Zhou H, Solomon NC, Fizazi K, . Meta-analysis evaluating the impact of site of metastasis on overall survival in men with castration-resistant prostate cancer. J Clin Oncol 2016;34:1652–9. 26951312 10.1200/JCO.2015.65.7270 PMC4872320

[48] Mehtälä J , Zong J, Vassilev Z, Brobert G, Gabarró MS, Stattin P, . Overall survival and second primary malignancies in men with metastatic prostate cancer. PLoS One 2020;15:e0227552. 32084147 10.1371/journal.pone.0227552 PMC7034858

[49] Tonry C , Finn S, Armstrong J, Pennington SR. Clinical proteomics for prostate cancer: understanding prostate cancer pathology and protein biomarkers for improved disease management. Clin Proteomics 2020;17:41. 33292167 10.1186/s12014-020-09305-7 PMC7678104

[50] Tsilidis KK , Markozannes G, Becerra-Tomás N, Cariolou M, Balducci K, Vieira R, . Post-diagnosis adiposity, physical activity, sedentary behaviour, dietary factors, supplement use and colorectal cancer prognosis: Global Cancer Update Programme (CUP Global) summary of evidence grading. Int J Cancer 2024;155:471–85. 38692587 10.1002/ijc.34904

[51] Bennett JP , Ford KL, Siervo M, Gonzalez MC, Lukaski HC, Sawyer MB, . Advancing body composition assessment in patients with cancer: first comparisons of traditional versus multicompartment models. Nutrition 2024;125:112494. 38843564 10.1016/j.nut.2024.112494

[52] Cantarutti A , Bonn SE, Adami HO, Grönberg H, Bellocco R, Bälter K. Body mass index and mortality in men with prostate cancer. Prostate 2015;75:1129–36. 25929695 10.1002/pros.23001

[53] Xu MC , Huelster HL, Hatcher JB, Avulova S, Stocks BT, Glaser ZA, . Obesity is associated with longer survival independent of sarcopenia and myosteatosis in metastatic and/or castrate-resistant prostate cancer. J Urol 2021;205:800–5. 33080148 10.1097/JU.0000000000001428 PMC10062423

[54] Vidal AC , Howard LE, de Hoedt A, Kane CJ, Terris MK, Aronson WJ, . Obese patients with castration-resistant prostate cancer may be at a lower risk of all-cause mortality: results from the Shared Equal Access Regional Cancer Hospital (SEARCH) database. BJU Int 2018;122:76–82. 29521009 10.1111/bju.14193 PMC5997525

[55] Martini A , Shah QN, Waingankar N, Sfakianos JP, Tsao CK, Necchi A, . The obesity paradox in metastatic castration-resistant prostate cancer. Prostate Cancer Prostatic Dis 2021;25:472–8. 34226662 10.1038/s41391-021-00418-0

[56] Farris MS , Courneya KS, Kopciuk KA, McGregor SE, Friedenreich CM. Anthropometric measurements and survival after a prostate cancer diagnosis. Br J Cancer 2018;118:607–10. 29235565 10.1038/bjc.2017.440 PMC5830594

[57] Jackson MD , Tulloch-Reid MK, McCaw-Binns AM, Aiken W, Ferguson TS, Bennett NR, . Central adiposity at diagnosis may reduce prostate cancer-specific mortality in African-Caribbean men with prostate cancer: 10-year follow-up of participants in a case-control study. Cancer Causes Control 2020;31:651–62. 32358695 10.1007/s10552-020-01306-z

[58] Dickerman BA , Ahearn TU, Giovannucci E, Stampfer MJ, Nguyen PL, Mucci LA, . Weight change, obesity and risk of prostate cancer progression among men with clinically localized prostate cancer. Int J Cancer 2017;141:933–44. 28543830 10.1002/ijc.30803 PMC5518616

[59] Davies BJ , Smaldone MC, Sadetsky N, Dall'era M, Carroll PR. The impact of obesity on overall and cancer specific survival in men with prostate cancer. J Urol 2009;182:112–7. 19447437 10.1016/j.juro.2009.02.118

[60] Polesel J , Gini A, Dal Maso L, Stocco C, Birri S, Taborelli M, . The impact of diabetes and other metabolic disorders on prostate cancer prognosis. J Diabetes Complications 2016;30:591–6. 26936307 10.1016/j.jdiacomp.2016.02.008

[61] Thomas EL , Parkinson JR, Frost GS, Goldstone AP, Doré CJ, McCarthy JP, . The missing risk: MRI and MRS phenotyping of abdominal adiposity and ectopic fat. Obesity (Silver Spring) 2012;20:76–87. 21660078 10.1038/oby.2011.142

[62] Champ CE , Francis L, Klement RJ, Dickerman R, Smith RP. Fortifying the treatment of prostate cancer with physical activity. Prostate Cancer 2016;2016:9462975. 26977321 10.1155/2016/9462975 PMC4764749

[63] Cao Y , Ma J. Body mass index, prostate cancer-specific mortality, and biochemical recurrence: a systematic review and meta-analysis. Cancer Prev Res (Phila) 2011;4:486–501. 21233290 10.1158/1940-6207.CAPR-10-0229 PMC3071449

[64] Zhong S , Yan X, Wu Y, Zhang X, Chen L, Tang J, . Body mass index and mortality in prostate cancer patients: a dose-response meta-analysis. Prostate Cancer Prostatic Dis 2016;19:122–31. 26754262 10.1038/pcan.2015.64

[65] Rivera-Izquierdo M , Pérez de Rojas J, Martínez-Ruiz V, Pérez-Gómez B, Sánchez MJ, Khan KS, . Obesity as a risk factor for prostate cancer mortality: a systematic review and dose-response meta-analysis of 280,199 patients. Cancers (Basel) 2021;13:4169. 34439328 10.3390/cancers13164169 PMC8392042

[66] Jin X , Qiu T, Li L, Yu R, Chen X, Li C, . Pathophysiology of obesity and its associated diseases. Acta Pharm Sin B 2023;13:2403–24. 37425065 10.1016/j.apsb.2023.01.012 PMC10326265

[67] Wilson RL , Taaffe DR, Newton RU, Hart NH, Lyons-Wall P, Galvão DA. Obesity and prostate cancer: a narrative review. Crit Rev Oncol Hematol 2022;169:103543. 34808374 10.1016/j.critrevonc.2021.103543

[68] Daniels JP , Freedland SJ, Gresham G. The growing implications of obesity for prostate cancer risk and mortality: where do we go from here?. J Natl Cancer Inst 2023;115:1448–50. 37587090 10.1093/jnci/djad140 PMC10699795

[69] Anderson AS , Martin RM, Renehan AG, Cade J, Copson ER, Cross AJ, . Cancer survivorship, excess body fatness and weight-loss intervention-where are we in 2020?. Br J Cancer 2020;124:1057–65. 33235316 10.1038/s41416-020-01155-2 PMC7961062

[70] Quail DF , Dannenberg AJ. The obese adipose tissue microenvironment in cancer development and progression. Nat Rev Endocrinol 2019;15:139–54. 30459447 10.1038/s41574-018-0126-x PMC6374176

[71] Stark T , Livas L, Kyprianou N. Inflammation in prostate cancer progression and therapeutic targeting. Transl Androl Urol 2015;4:455–63. 26816843 10.3978/j.issn.2223-4683.2015.04.12 PMC4708587

[72] Howe LR , Subbaramaiah K, Hudis CA, Dannenberg AJ. Molecular pathways: adipose inflammation as a mediator of obesity-associated cancer. Clin Cancer Res 2013;19:6074–83. 23958744 10.1158/1078-0432.CCR-12-2603 PMC3891839

[73] Fujita K , Hayashi T, Matsushita M, Uemura M, Nonomura N. Obesity, inflammation, and prostate cancer. J Clin Med 2019;8:201. 30736371 10.3390/jcm8020201 PMC6406330

[74] Slawinski CGV , Barriuso J, Guo H, Renehan AG. Obesity and cancer treatment outcomes: interpreting the complex evidence. Clin Oncol (R Coll Radiol) 2020;32:591–608. 32595101 10.1016/j.clon.2020.05.004

[75] Langlais CS , Cowan JE, Neuhaus J, Kenfield SA, Van Blarigan EL, Broering JM, . Obesity at diagnosis and prostate cancer prognosis and recurrence risk following primary treatment by radical prostatectomy. Cancer Epidemiol Biomarkers Prev 2019;28:1917–25. 31462398 10.1158/1055-9965.EPI-19-0488 PMC6825577

[76] Allott EH , Masko EM, Freedland SJ. Obesity and prostate cancer: weighing the evidence. Eur Urol 2013;63:800–9. 23219374 10.1016/j.eururo.2012.11.013 PMC3597763

[77] Hespe GE , Kataru RP, Savetsky IL, García Nores GD, Torrisi JS, Nitti MD, . Exercise training improves obesity-related lymphatic dysfunction. J Physiol 2016;594:4267–82. 26931178 10.1113/JP271757 PMC4967732

[78] Nitti MD , Hespe GE, Kataru RP, García Nores GD, Savetsky IL, Torrisi JS, . Obesity-induced lymphatic dysfunction is reversible with weight loss. J Physiol 2016;594:7073–87. 27619475 10.1113/JP273061 PMC5134379

[79] Schulze MB , Martínez-González MA, Fung TT, Lichtenstein AH, Forouhi NG. Food based dietary patterns and chronic disease prevention. BMJ 2018;361:k2396. 29898951 10.1136/bmj.k2396 PMC5996879

[80] Ording AG , Cronin-Fenton D, Ehrenstein V, Lash TL, Acquavella J, Rørth M, . Challenges in translating endpoints from trials to observational cohort studies in oncology. Clin Epidemiol 2016;8:195–200. 27354827 10.2147/CLEP.S97874 PMC4910679

[81] Archer M , Dogra N, Kyprianou N. Inflammation as a driver of prostate cancer metastasis and therapeutic resistance. Cancers 2020;12:2984. 33076397 10.3390/cancers12102984 PMC7602551

[82] Lewis L , Thompson B, Stellmaker R, Koelmeyer L. Body composition and chemotherapy toxicities in breast cancer: a systematic review of the literature. J Cancer Surviv 2024;19:914–29. 38206431 10.1007/s11764-023-01512-z PMC12081505

[83] Feng Q , Bešević J, Conroy M, Omiyale W, Lacey B, Allen N. Comparison of body composition measures assessed by bioelectrical impedance analysis versus dual-energy X-ray absorptiometry in the United Kingdom Biobank. Clin Nutr ESPEN 2024;63:214–25. 38970786 10.1016/j.clnesp.2024.06.040

[84] Chubak J , Boudreau DM, Wirtz HS, McKnight B, Weiss NS. Threats to validity of nonrandomized studies of postdiagnosis exposures on cancer recurrence and survival. J Natl Cancer Inst 2013;105:1456–62. 23940288 10.1093/jnci/djt211 PMC3787908

[85] Kiss N , Prado CM, Daly RM, Denehy L, Edbrooke L, Baguley BJ, . Low muscle mass, malnutrition, sarcopenia, and associations with survival in adults with cancer in the UK Biobank cohort. J Cachexia Sarcopenia Muscle 2023;14:1775–88. 37212184 10.1002/jcsm.13256 PMC10401543

[86] Monroy-Iglesias MJ , Russell B, Crawley D, Allen NE, Travis RC, Perez-Cornago A, . Metabolic syndrome biomarkers and prostate cancer risk in the UK Biobank. Int J Cancer 2021;148:825–34. 33405276 10.1002/ijc.33255

[87] Nevedomskaya E , Baumgart SJ, Haendler B. Recent advances in prostate cancer treatment and drug discovery. Int J Mol Sci 2018;19:1359. 29734647 10.3390/ijms19051359 PMC5983695

[88] Fritz J , Jochems SHJ, Bjørge T, Wood AM, Häggström C, Ulmer H, . Body mass index, triglyceride-glucose index, and prostate cancer death: a mediation analysis in eight European cohorts. Br J Cancer 2024;130:308–16. 38087039 10.1038/s41416-023-02526-1 PMC10803806

[89] Polesel J , Gini A, Dal Maso L, Stocco C, Birri S, Taborelli M, . The negative impact of tobacco smoking on survival after prostate cancer diagnosis. Cancer Causes Control 2015;26:1299–305. 26134048 10.1007/s10552-015-0624-2

[90] Park Y , Peterson LL, Colditz GA. The plausibility of obesity paradox in cancer-point. Cancer Res 2018;78:1898–903. 29654151 10.1158/0008-5472.CAN-17-3043 PMC5903573

