# Supplementary material for: Adiposity Status Close to Diagnosis and Its Association with Prostate Cancer Survival in the UK Biobank
Source: Cancer Res Commun. 2025 Jul 16;5(7):1155–70. doi: 10.1158/2767-9764.CRC-25-0124 (PMC12264726; doi:10.1158/2767-9764.CRC-25-0124)

**Supplementary Figure 1 – Directed acyclic graph showing potential causal relations between the variables considered in the present study.**


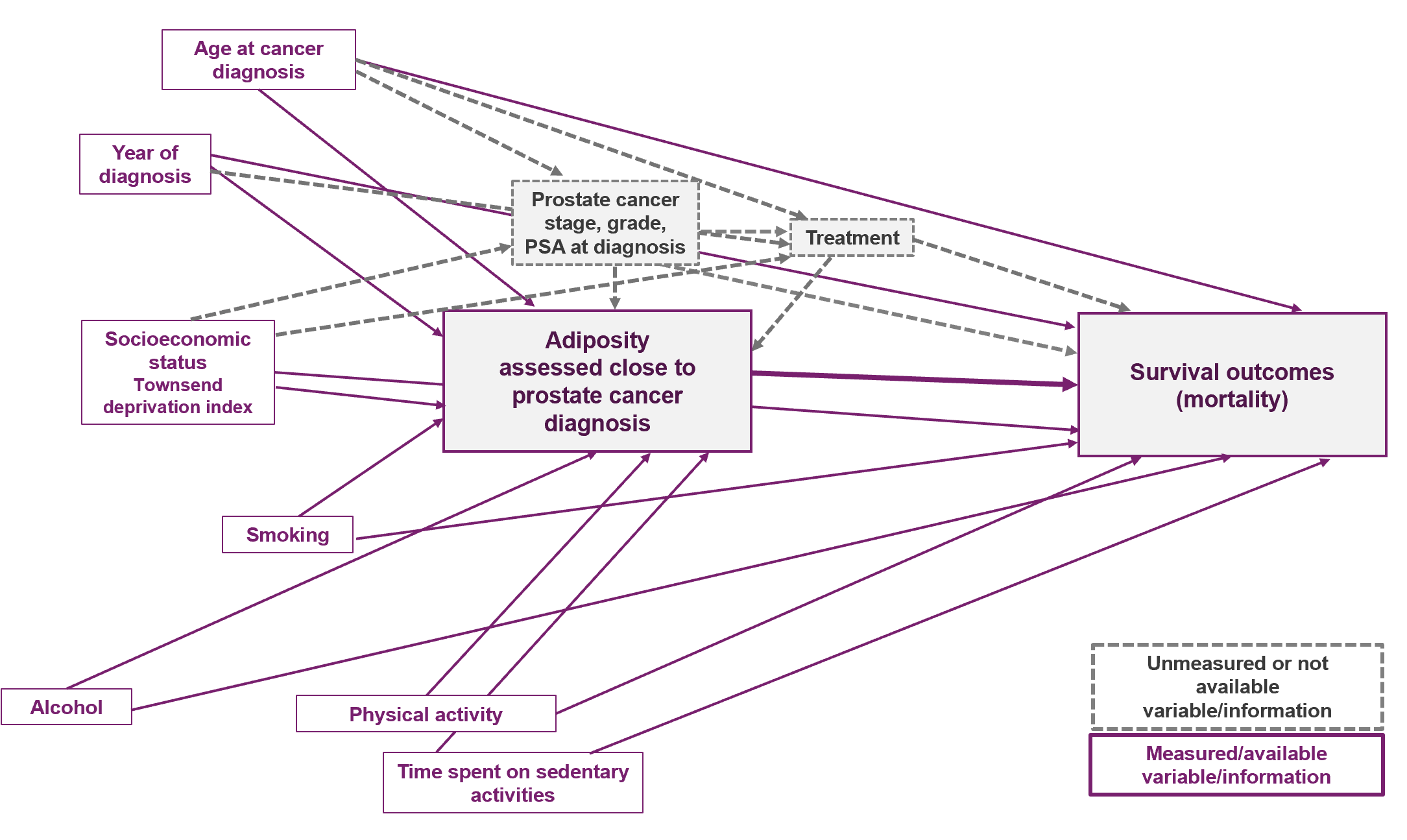

Supplement: Supplementary Figure 1 — Directed acyclic graph showing potential causal relations between the variables considered in the present study. [file crc-25-0124_supplementary_figure_1_suppsf1.docx]
