# Supplementary material for: Adiposity Status Close to Diagnosis and Its Association with Prostate Cancer Survival in the UK Biobank
Source: Cancer Res Commun. 2025 Jul 16;5(7):1155–70. doi: 10.1158/2767-9764.CRC-25-0124 (PMC12264726; doi:10.1158/2767-9764.CRC-25-0124)
Supplement: Supplementary Figure 3 — Restricted cubic spline analysis for hip circumference assessed close to diagnosis and all-cause, prostate cancer-specific and non-prostate cancer mortality. [file crc-25-0124_supplementary_figure_3_suppsf3.docx]

**Supplementary Figure 3 *–* Restricted cubic spline analysis for hip circumference assessed close to diagnosis and all-cause, prostate cancer-specific and non-prostate cancer mortality.**


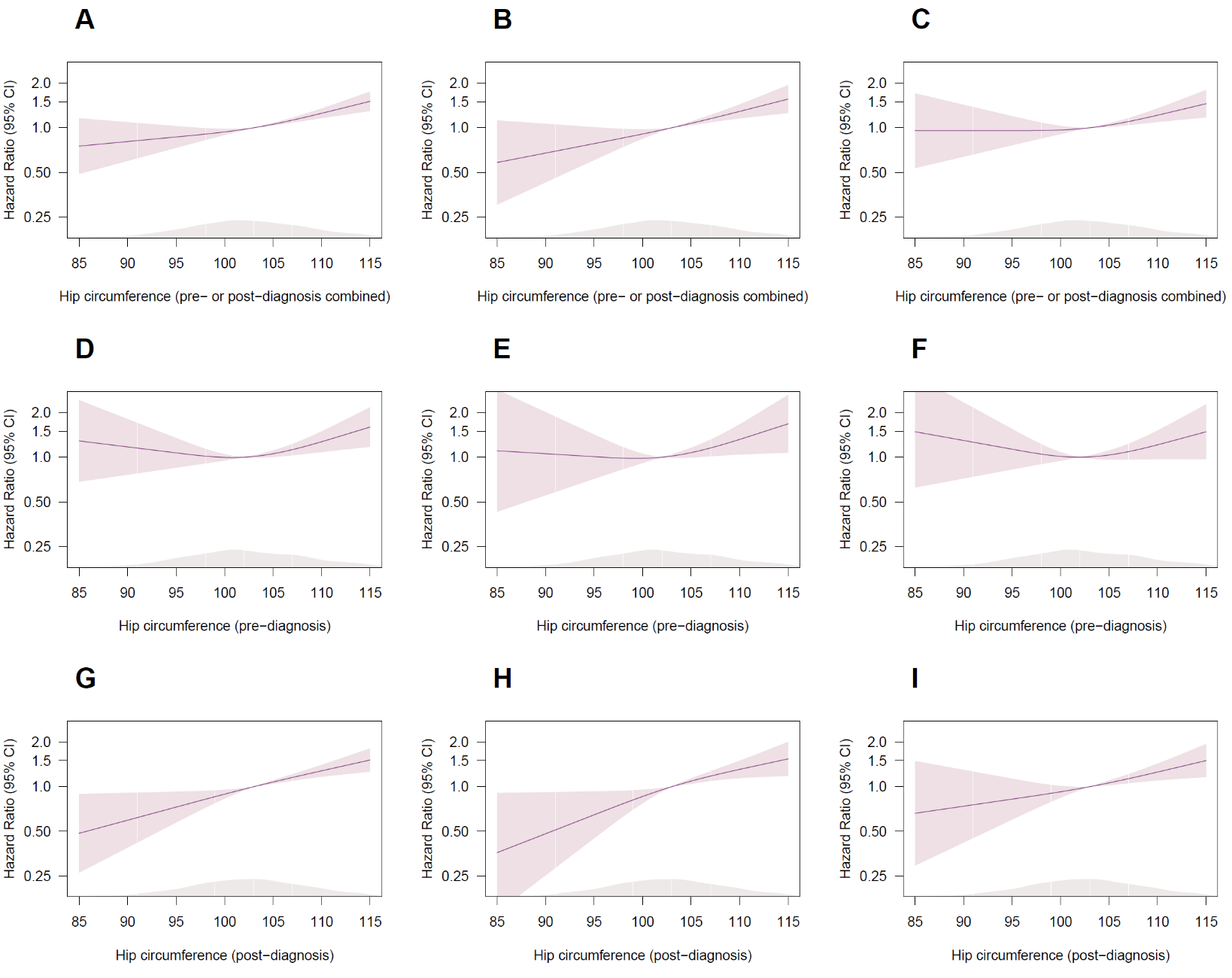
Hazard ratios (HRs) from Cox proportional hazards model with restricted cubic spline curves describing the association between hip circumference (cm) collected pre- or post-diagnosis combined and **A)** all-cause mortality (deaths=680) **B)** prostate cancer-specific mortality (deaths=331), **C)** non-prostate cancer mortality (deaths=347); pre-diagnosis hip circumference and **D)** all-cause mortality (deaths=262), **E)** prostate cancer-specific mortality (deaths=124), **F)** non-prostate cancer mortality (deaths=137); post-diagnosis hip circumference and **G)** all-cause mortality (deaths=418), **H)** prostate cancer-specific mortality (deaths=207), **I)** non-prostate cancer mortality (deaths=210). HRs are based on the main model adjusted for age of diagnosis, year of diagnosis, smoking status, physical activity, sedentary activities, Townsend deprivation index, alcohol intake frequency; knots at the 10th, 50^th^ and 90^th^ percentiles of hip circumference. The median hip circumference of the individuals included in analyses was used as referent: 103 cm in the pre- or post-diagnosis analysis, 102 cm in the pre-diagnosis analysis and 103 cm in the post-diagnosis analysis. The smooth density plot represents the density of the population across the spline variable.
