# Supplementary material for: Adiposity Status Close to Diagnosis and Its Association with Prostate Cancer Survival in the UK Biobank
Source: Cancer Res Commun. 2025 Jul 16;5(7):1155–70. doi: 10.1158/2767-9764.CRC-25-0124 (PMC12264726; doi:10.1158/2767-9764.CRC-25-0124)
Supplement: Supplementary Table 1 — Relevant covariates available in UK Biobank database based on subject matter knowledge. [file crc-25-0124_supplementary_table_1_suppst1.docx]

| **Supplementary Table 1 – Relevant covariates available in UK Biobank database based on subject matter knowledge.** | | |
| --- | --- | --- |
| Confounder | Relevance to obesity | Relevance to mortality (survival) |
| Age | Due to increased life expectancy obesity is more prevalent among older age groups. Ageing is associated with increased abdominal white adipose tissue and fat deposition in skeletal muscle.^1^ Lifestyle changes in the elderly can result in excess fat tissue accumulation, that in turn accelerates the development of age-related diseases.^2^ | Mortality increases with age (has shown to double every 6-7 years during adulthood).^3^ Prostate cancer mortality rates strongly associated with age.^4^ |
| Year of diagnosis | Obesity prevalence has doubled worldwide since 1980^5^ and increased four-fold in men between 1975 and 2016^6^. Projections indicate that obesity is expected to reach maximal levels for men between 2030 and 2052.^7^ | Prostate cancer detection, treatment, and survival outcomes have improved with time.^8-10^ |
| Smoking | Quitting smoking has been associated with weight gain.^11^ There is some evidence that high tobacco consumption may lower body weight.^12^ | Smoking increases risk of aggressive prostate cancer, all-cause and prostate cancer-specific mortality.^13-16^ |
| Physical activity | Increased physical activity associated with weight loss.^17^ | Physical activity has been associated with lower risk of all-cause and prostate cancer-specific mortality.^18^ |
| Townsend deprivation index (as proxy for socioeconomic status) | Socio-economic status is an important determinant of obesity development in adults.^19, 20^ | Lower education and socio-economic status may increase risk of death in prostate cancer patients.^21^ |
| Alcohol consumption | Light-to-moderate drinkers have on average lower BMI and WHR and are less likely to be obese and to develop type 2 diabetes compared to non-drinkers and heavy drinkers.^22^ Evidence from observational studies showed non-linear trends of the associations between alcohol consumption and obesity outcomes although these could have been confounded by other lifestyle behaviours.^22^ | Alcohol consumption could influence disease progression (could be a modifiable factor for cancer survival).^23^ The biologic impact of alcohol is complex; its effect could vary according to the exposure level. Abstinence was associated with a higher risk of prostate cancer-specific mortality versus light alcohol consumption. ^24^ |
| **References**  1. Jura M, Kozak LP. Obesity and related consequences to ageing. *Age (Dordr)*. 2016;**38**(1):23.  2. Tchkonia T, Morbeck DE, Von Zglinicki T, Van Deursen J, Lustgarten J, Scrable H. et al. Fat tissue, aging, and cellular senescence. *Aging Cell*. 2010;**9**(5):667-84.  3. Ebeling M, Rau R, Malmström H, Ahlbom A, Modig K. The rate by which mortality increase with age is the same for those who experienced chronic disease as for the general population. *Age Ageing*. 2021;**50**(5):1633-40.  4. Rawla P. Epidemiology of Prostate Cancer. *World J Oncol*. 2019;**10**(2):63-89.  5. Fujita K, Hayashi T, Matsushita M, Uemura M, Nonomura N. Obesity, Inflammation, and Prostate Cancer. *J Clin Med*. 2019;**8**(2).  6. Sung H, Siegel RL, Torre LA, Pearson-Stuttard J, Islami F, Fedewa SA. et al. Global patterns in excess body weight and the associated cancer burden. *CA Cancer J Clin*. 2019;**69**(2):88-112.  7. Janssen F, Bardoutsos A, Vidra N. Obesity Prevalence in the Long-Term Future in 18 European Countries and in the USA. *Obes Facts*. 2020;**13**(5):514-27.  8. Kim MM, Hoffman KE, Levy LB, Frank SJ, Pugh TJ, Choi S. et al. Improvement in prostate cancer survival over time: a 20-year analysis. *Cancer J*. 2012;**18**(1):1-8.  9. Barsouk A, Padala SA, Vakiti A, Mohammed A, Saginala K, Thandra KC. et al. Epidemiology, Staging and Management of Prostate Cancer. *Med Sci (Basel)*. 2020;**8**(3).  10. Nevedomskaya E, Baumgart SJ, Haendler B. Recent Advances in Prostate Cancer Treatment and Drug Discovery. *Int J Mol Sci*. 2018;**19**(5).  11. Tian J, Venn A, Otahal P, Gall S. The association between quitting smoking and weight gain: a systematic review and meta-analysis of prospective cohort studies. *Obes Rev*. 2015;**16**(10):883-901.  12. Winsløw UC, Rode L, Nordestgaard BG. High tobacco consumption lowers body weight: a Mendelian randomization study of the Copenhagen General Population Study. *Int J Epidemiol*. 2015;**44**(2):540-50.  13. Müezzinler A, Mons U, Gellert C, Schöttker B, Jansen E, Kee F. et al. Smoking and All-cause Mortality in Older Adults: Results From the CHANCES Consortium. *Am J Prev Med*. 2015;**49**(5):e53-e63.  14. Langlais CS, Graff RE, Van Blarigan EL, Palmer NR, Washington SL, 3rd, Chan JM. et al. Post-Diagnostic Dietary and Lifestyle Factors and Prostate Cancer Recurrence, Progression, and Mortality. *Curr Oncol Rep*. 2021;**23**(3):37.  15. Moreira DM, Aronson WJ, Terris MK, Kane CJ, Amling CL, Cooperberg MR. et al. Cigarette smoking is associated with an increased risk of biochemical disease recurrence, metastasis, castration-resistant prostate cancer, and mortality after radical prostatectomy: results from the SEARCH database. *Cancer*. 2014;**120**(2):197-204.  16. Kenfield SA, Stampfer MJ, Chan JM, Giovannucci E. Smoking and prostate cancer survival and recurrence. *JAMA*. 2011;**305**(24):2548-55.  17. Chin SH, Kahathuduwa CN, Binks M. Physical activity and obesity: what we know and what we need to know. *Obes Rev*. 2016;**17**(12):1226-44.  18. Friedenreich CM, Stone CR, Cheung WY, Hayes SC. Physical Activity and Mortality in Cancer Survivors: A Systematic Review and Meta-Analysis. *JNCI Cancer Spectr*. 2019;**4**(1):pkz080.  19. Sacks G, Swinburn B, Lawrence M. Obesity Policy Action framework and analysis grids for a comprehensive policy approach to reducing obesity. *Obes Rev*. 2009;**10**(1):76-86.  20. Masood B, Moorthy M. Causes of obesity: a review. *Clin Med (Lond)*. 2023;**23**(4):284-91.  21. DeRouen MC, Schupp CW, Koo J, Yang J, Hertz A, Shariff-Marco S. et al. Impact of individual and neighborhood factors on disparities in prostate cancer survival. *Cancer Epidemiol*. 2018;**53**:1-11.  22. Lu T, Nakanishi T, Yoshiji S, Butler-Laporte G, Greenwood CMT, Richards JB. Dose-dependent Association of Alcohol Consumption With Obesity and Type 2 Diabetes: Mendelian Randomization Analyses. *J Clin Endocrinol Metab*. 2023;**108**(12):3320-9.  23. Brunner C, Davies NM, Martin RM, Eeles R, Easton D, Kote-Jarai Z. et al. Alcohol consumption and prostate cancer incidence and progression: A Mendelian randomisation study. *Int J Cancer*. 2017;**140**(1):75-85.  24. Dickerman BA, Markt SC, Koskenvuo M, Pukkala E, Mucci LA, Kaprio J. Alcohol intake, drinking patterns, and prostate cancer risk and mortality: a 30-year prospective cohort study of Finnish twins. *Cancer Causes Control*. 2016;**27**(9):1049-58. | | |
