# Supplementary material for: Adiposity Status Close to Diagnosis and Its Association with Prostate Cancer Survival in the UK Biobank
Source: Cancer Res Commun. 2025 Jul 16;5(7):1155–70. doi: 10.1158/2767-9764.CRC-25-0124 (PMC12264726; doi:10.1158/2767-9764.CRC-25-0124)
Supplement: Supplementary Table 2 — Missing covariate data from the 3,916 men (prior to elimination of individuals with missing data). [file crc-25-0124_supplementary_table_2_suppst2.docx]

| **Supplementary Table 2 – Missing covariate data from the 3,916 men (prior to elimination of individuals with missing data).** | | |  |  |
| --- | --- | --- | --- | --- |
| **% data missing^a^** | **Adiposity close to diagnosis**  **(pre-or post-diagnosis combined analysis)** | |  |  |
| Covariate | % | N (out of 3,916) |  |  |
| Sum of physical activity (walking, moderate, vigorous) excess MET-hours/week | 3% | 131 |  |  |
| Smoking status | <1% | 32 |  |  |
| Sedentary activities (hours/day) | <1% | 13 |  |  |
| Townsend deprivation index | <1% | <10 |  |  |
| Alcohol intake frequency | <1% | 10 |  |  |
| **^a^**Year of diagnosis and age of diagnosis: no missing data. Similar % missingness in the separate pre- and post-diagnosis analyses. No available data/information on stage, grade, treatment, PSA. | | |  |  |
