# Supplementary material for: Adiposity Status Close to Diagnosis and Its Association with Prostate Cancer Survival in the UK Biobank
Source: Cancer Res Commun. 2025 Jul 16;5(7):1155–70. doi: 10.1158/2767-9764.CRC-25-0124 (PMC12264726; doi:10.1158/2767-9764.CRC-25-0124)
Supplement: Supplementary Table 3 — Important demographic characteristics of the 3,760 men with prostate cancer in UK Biobank according to the BMI, WHO categories. [file crc-25-0124_supplementary_table_3_suppst3.docx]

| **Supplementary Table 3 – Important demographic characteristics of the 3,760 men with prostate cancer in UK Biobank according to the BMI, WHO categories.** | | | |
| --- | --- | --- | --- |
|  | **Underweight & Normal weight**  (BMI≤24.9)  N=926 | **Overweight**  (BMI: 25-29.9)  N=1,938 | **Obese**  (BMI≥30)  N=896 |
| *Follow-up time (time from return of either baseline or each respective follow-up measurement until death/censoring), median years (p2.5-p97.5)* | 11.5 (2.1-13.4) | 11.4 (1.7-13.3) | 11.2 (1.7-13.2) |
| *Age at diagnosis, years, median (p2.5-p97.5)* | 64 (53-72) | 64 (52-71) | 63 (54-71) |
| *Smoking status* |  |  |  |
| Never smoker, n (%) | 515 (56) | 914 (47) | 359 (40) |
| Previous smoker, n (%) | 340 (37) | 881 (45) | 466 (52) |
| Current smoker, n (%) | 66 (7) | 143 (7) | 71 (8) |
| ***^a^*** *Physical activity (excess MET-hours/week), median (p2.5-p97.5)* | 22.7 (0-125.6) | 21.8 (0.0-137.0) | 16.1 (0.0-140.2) |
| ***^b^****Sedentary activities (hours/day)* | 5.0 (2.0-9.0) | 5.0 (3.0-9.0) | 6.0 (3.0-10.0) |
| *Townsend deprivation index, median (p2.5-p97.5)* | -2.5 (-5.3 to 5.4) | -2.6 (-5.6 to 5.4) | -2.2 (-5.4 to 6.2) |
| *Alcohol intake frequency* |  |  |  |
| Never, n (%) | 71 (8) | 107 (6) | 50 (6) |
| Special occasions only, n (%) | 59 (6) | 145 (7) | 89 (10) |
| One to three times monthly, n (%) | 71 (8) | 152 (8) | 89 (10) |
| Once or twice weekly, n (%) | 210 (23) | 465 (24) | 248 (28) |
| Three or four times weekly, n (%) | 244 (26) | 508 (26) | 199 (22) |
| Daily or almost daily, n (%) | 271 (29) | 561 (29) | 221 (25) |
| **^a^** Physical activity as the sum of walking, moderate and vigorous activities in excess MET-hours/week.  **^b^** Sedentary activities as the sum of total time spent watching television, using a computer screen or driving in hours/day. | | | |
