# Supplementary material for: Adiposity Status Close to Diagnosis and Its Association with Prostate Cancer Survival in the UK Biobank
Source: Cancer Res Commun. 2025 Jul 16;5(7):1155–70. doi: 10.1158/2767-9764.CRC-25-0124 (PMC12264726; doi:10.1158/2767-9764.CRC-25-0124)
Supplement: Supplementary Table 4 — Categorical analysis according to the BMI WHO categories and mortality. [file crc-25-0124_supplementary_table_4_suppst4.docx]

| **Supplementary Table 4 – Categorical analysis according to the BMI WHO categories and mortality.** | | | | | | |
| --- | --- | --- | --- | --- | --- | --- |
|  | **Pre- or post-diagnosis BMI** | | **Post-diagnosis BMI** | | **Pre-diagnosis BMI** | |
|  | **N_e/_N_t_** | **HR^a^ (95% CI)** | **N_e/_N_t_** | **HR^a^ (95% CI)** | **N_e/_N_t_** | **HR^a^ (95% CI)** |
| *All-cause mortality* |  |  |  |  |  |  |
| Normal weight (≤24.9 kg/m^2^) **^b^** | 131/926 | 1 | 68/566 | 1 | 63/360 | 1 |
| Overweight (25-29.9 kg/m^2^) | 331/1,938 | 1.24 (1.01-1.52) | 194/1215 | 1.32 (1.00-1.74) | 137/723 | 1.22 (0.89-1.66) |
| Obese (≥30 kg/m^2^) | 218/896 | 1.80 (1.44-2.25) | 156/589 | 2.10 (1.56-2.82) | 62/307 | 1.53 (1.05-2.24) |
| *Prostate cancer-specific mortality* | | | | | | |
| Normal weight^2^ (≤24.9 kg/m^2^) **^b^** | 59/926 | 1 | 28/566 | 1 | 31/360 | 1 |
| Overweight (25-29.9 kg/m^2^) | 170/1,938 | 1.41 (1.04-1.90) | 103/1215 | 1.69 (1.11-2.58) | 67/723 | 1.21 (0.78-1.88) |
| Obese (≥30 kg/m^2^) | 102/896 | 1.89 (1.36-2.63) | 76/589 | 2.52 (1.61-3.94) | 26/307 | 1.27 (0.72-2.22) |
| **^a^** Model adjusted for: age of diagnosis (continuous), year of diagnosis (continuous), smoking status (categorical), physical activity as sum of excess MET-hours/week for walking, moderate and vigorous activities (continuous), sedentary activities as sum of total time spent watching television, using a computer screen or driving in hours/day. Stratified by UK Biobank participating centres. Survival time estimated in days as the difference between the date of censoring or death minus the date of questionnaire (matched to the period that each specific case was selected from in our analytic subset).  **^b^** The normal weight category also includes men with underweight as the number of individuals with underweight was very small (N=7 total with pre- or post-diagnosis BMI, N=3 with post-diagnosis BMI and N=4 with pre-diagnosis BMI).  Abbreviations: BMI, Body mass index; N_e_, Number of events; N_t_, Total number of men, WHO, World Health Organisation; | | | | | | |
