# Supplementary material for: Adiposity Status Close to Diagnosis and Its Association with Prostate Cancer Survival in the UK Biobank
Source: Cancer Res Commun. 2025 Jul 16;5(7):1155–70. doi: 10.1158/2767-9764.CRC-25-0124 (PMC12264726; doi:10.1158/2767-9764.CRC-25-0124)
Supplement: Supplementary Table 5 — Sensitivity analysis, in each year of adiposity measurement. Cox Proportional HRs and 95%CIs for the linear association between adiposity by each year of adiposity measurement and all-cause and prostate cancer-specific mortality. [file crc-25-0124_supplementary_table_5_suppst5.docx]

| **Supplementary Table 5 – Sensitivity analysis, in each year of adiposity measurement. Cox Proportional HRs and 95%CIs for the linear association between adiposity by each year of adiposity measurement and all-cause and prostate cancer-specific mortality.** | | | | |
| --- | --- | --- | --- | --- |
|  | **All-cause mortality** | | **Prostate cancer-specific mortality** | |
|  | **N_e/_N_t_** | **HR^a^ (95%CI)** | **N_e/_N_t_** | **HR^a^ (95%CI)** |
| **By each year of BMI measurement (up to two years pre-diagnosis and up to five years post-diagnosis), per 5 kg/m^2^** | | | | |
| First year pre-diagnosis | 130/662 | 1.24 (0.98-1.58) | 60/662 | 1.21 (0.84-1.72) |
|  |  |  |  |  |
| Second year pre-diagnosis | 132/728 | 1.29 (1.01-1.66) | 64/728 | 1.16 (0.82-1.66) |
|  |  |  |  |  |
| First year post-diagnosis | 94/540 | 1.58 (1.19-2.10) | 45/540 | 1.46 (0.97-2.19) |
|  |  |  |  |  |
| Second year post-diagnosis | 107/569 | 1.37 (1.05-1.80) | 53/569 | 1.75 (1.17-2.62) |
|  |  |  |  |  |
| Third year post-diagnosis | 73/444 | 1.42 (1.04-1.93) | 38/444 | 1.62 (1.07-2.47) |
|  |  |  |  |  |
| Fourth year post-diagnosis | 71/437 | 1.40 (0.99-1.96) | 36/437 | 1.44 (0.83-2.48) |
|  |  |  |  |  |
| Fifth year post-diagnosis | 73/380 | 1.41 (1.02-1.95) | 35/380 | 1.76 (1.05-2.93) |
| **By each year of waist circumference measurement (up to two years pre-diagnosis and up to five years post-diagnosis), per 10 cm** | | | | |
| First year pre-diagnosis | 130/662 | 1.29 (1.08-1.55) | 60/662 | 1.36 (1.04-1.77) |
|  |  |  |  |  |
| Second year pre-diagnosis | 132/728 | 1.30 (1.08-1.55) | 64/728 | 1.25 (0.97-1.61) |
|  |  |  |  |  |
| First year post-diagnosis | 94/540 | 1.29 (1.04-1.58) | 45/540 | 1.16 (0.86-1.57) |
|  |  |  |  |  |
| Second year post-diagnosis | 107/569 | 1.43 (1.17-1.75) | 53/569 | 1.76 (1.30-2.38) |
|  |  |  |  |  |
| Third year post-diagnosis | 73/444 | 1.24 (0.99-1.57) | 38/444 | 1.35 (0.99-1.86) |
|  |  |  |  |  |
| Fourth year post-diagnosis | 71/437 | 1.33 (1.04-1.72) | 36/437 | 1.35 (0.74-2.02) |
|  |  |  |  |  |
| Fifth year post-diagnosis | 73/380 | 1.34 (1.05-1.71) | 35/380 | 1.45 (0.99-2.12) |
| **By each year of hip circumference measurement (up to two years pre-diagnosis and up to five years post-diagnosis), per 10 cm** | | | | |
| First year pre-diagnosis | 130/662 | 1.39 (1.06-1.82) | 60/662 | 1.44 (0.97-2.14) |
|  |  |  |  |  |
| Second year pre-diagnosis | 132/728 | 1.12 (0.86-1.46) | 64/728 | 1.17 (0.80-1.71) |
|  |  |  |  |  |
| First year post-diagnosis | 94/540 | 1.37 (1.00-1.88) | 45/540 | 1.22 (0.78-1.91) |
|  |  |  |  |  |
| Second year post-diagnosis | 107/569 | 1.45 (1.07-1.95) | 53/569 | 1.81 (1.13-2.74) |
|  |  |  |  |  |
| Third year post-diagnosis | 73/444 | 1.64 (1.22-2.20) | 38/444 | 1.97 (1.31-2.97) |
|  |  |  |  |  |
| Fourth year post-diagnosis | 71/437 | 1.29 (0.88-1.89) | 36/437 | 1.35 (0.74-2.43) |
|  |  |  |  |  |
| Fifth year post-diagnosis | 73/380 | 1.47 (1.04-2.06) | 35/380 | 1.54 (0.89-2.67) |
| **By each year of waist-to-hip ratio measurement (up to two years pre-diagnosis and up to five years post-diagnosis), per 0.1 unit** | | | | |
| First year pre-diagnosis | 130/662 | 1.36 (1.01-1.83) | 60/662 | 1.52 (0.98-2.34) |
|  |  |  |  |  |
| Second year pre-diagnosis | 132/728 | 1.72 (1.27-2.33) | 64/728 | 1.50 (0.98-2.32) |
|  |  |  |  |  |
| First year post-diagnosis | 94/540 | 1.34 (0.94-1.90) | 45/540 | 1.17 (0.69-1.99) |
|  |  |  |  |  |
| Second year post-diagnosis | 107/569 | 1.64 (1.19-2.25) | 53/569 | 2.09 (1.31-3.30) |
|  |  |  |  |  |
| Third year post-diagnosis | 73/444 | 0.87 (0.59-1.29) | 38/444 | 0.81 (0.47-1.40) |
|  |  |  |  |  |
| Fourth year post-diagnosis | 71/437 | 1.70 (1.09-2.68) | 36/437 | 1.59 (0.81-3.14) |
|  |  |  |  |  |
| Fifth year post-diagnosis | 73/380 | 1.43 (0.94-2.18) | 35/380 | 1.56 (0.82-2.98) |
| **^a^** Models adjusted for: age of diagnosis, year of diagnosis, smoking status (categorical as: never, current, previous), physical activity (continuous as sum of excess MET-hours/week of walking, moderate and vigorous activity), sedentary activities (continuous as sum of time spent watching TV, using a computer screen, or driving in hours/day), Townsend deprivation index (in quintiles) and alcohol intake frequency (categorical as: never, special occasions only, one to three times monthly, once or twice weekly, daily or almost daily). Models stratified by UK Biobank centre. The date of each respective assessment visit (according to the period that the individual was selected from) was considered as the start of follow-up (entry-time). The date of death or censoring (31^st^ Dec 2020) was considered as the end of follow-up (exit-time).  Abbreviations: BMI, Body mass index; CI, Confidence Interval; HR, Hazard Ratio; N_e,_ number of events; N_t,_ total number of men with prostate cancer. | | | | |
