# Supplementary material for: Adiposity Status Close to Diagnosis and Its Association with Prostate Cancer Survival in the UK Biobank
Source: Cancer Res Commun. 2025 Jul 16;5(7):1155–70. doi: 10.1158/2767-9764.CRC-25-0124 (PMC12264726; doi:10.1158/2767-9764.CRC-25-0124)
Supplement: Supplementary Table 6 — Cox Proportional Hazard Ratios and 95%CIs for the linear association between adiposity close to diagnosis (pre- or post-diagnosis adiposity combined) and all-cause and prostate cancer-specific mortality in individuals with incident versus prevalent prostate cancer (fully adjusted/main model). [file crc-25-0124_supplementary_table_6_suppst6.docx]

| **Supplementary Table 6 – Cox Proportional Hazard Ratios and 95%CIs for the linear association between adiposity close to diagnosis (pre- or post-diagnosis adiposity combined) and all-cause and prostate cancer-specific mortality in individuals with incident versus prevalent prostate cancer (fully adjusted/main model).** | | | | |
| --- | --- | --- | --- | --- |
|  | **N_e/_N_t_** | **HR^a^ (95% CI)** | **N_e/_N_t_** | **HR^a^ (95% CI)** |
|  | **Incident prostate cancer** | | **Prevalent prostate cancer** | |
| **BMI (per 5 kg/m^2^)** |  |  |  |  |
|  |  |  |  |  |
| All-cause mortality: | 275/1,672 | 1.31 (1.11-1.54) | 405/2,088 | 1.34 (1.18-1.51) |
|  |  |  |  |  |
| Prostate cancer-specific mortality: | 132/1,672 | 1.29 (1.01-1.63) | 199/2,088 | 1.36 (1.14-1.63) |
|  |  |  |  |  |
| **Waist circumference (per 10 cm)** |  |  |  |  |
| All-cause mortality: | 275/1,672 | 1.28 (1.14-1.45) | 405/2,088 | 1.29 (1.17-1.41) |
|  |  |  |  |  |
| Prostate cancer-specific mortality: | 132/1,672 | 1.31 (1.10-1.55) | 199/2,088 | 1.31 (1.14-1.49) |
|  |  |  |  |  |
| **Hip circumference (per 10 cm)** |  |  |  |  |
| All-cause mortality: | 275/1,672 | 1.27 (1.06-1.52) | 405/2,088 | 1.40 (1.23-1.59) |
|  |  |  |  |  |
| Prostate cancer-specific mortality: | 132/1,672 | 1.35 (1.04-1.74) | 199/2,088 | 1.46 (1.21-1.76) |
|  |  |  |  |  |
| **Waist-to-hip ratio (per 0.1 unit)** |  |  |  |  |
| All-cause mortality: | 275/1,672 | 1.45 (1.19-1.77) | 405/2,088 | 1.31 (1.12-1.54) |
|  |  |  |  |  |
| Prostate cancer-specific mortality: | 132/1,672 | 1.45 (1.09-1.94) | 199/2,088 | 1.30 (1.03-1.64) |
|  |  |  |  |  |
| **^a^**Models adjusted for: age of diagnosis, year of diagnosis, smoking status (categorical as: never, current, previous), physical activity (continuous as sum of excess MET-hours/week of walking, moderate and vigorous activity), sedentary activities (continuous as sum of time spent watching TV, using a computer screen, or driving in hours/day), Townsend deprivation index (in quintiles) and alcohol intake frequency (categorical as: never, special occasions only, one to three times monthly, once or twice weekly, daily or almost daily).  Models stratified by UK Biobank centre. The date of each respective assessment visit (according to the period that the individual was selected from) was considered as the start of follow-up (entry-time). The date of death or censoring (31^st^ Dec 2020) was considered as the end of follow-up (exit-time). For the prevalent prostate cancer all the men were from the first post-diagnosis period.  Abbreviations: BMI, Body mass index; CI, Confidence Interval; HR, Hazard Ratio; N_e,_ number of events; N_t,_ total number of men with prostate cancer. | | | | |
