# Supplementary material for: Adiposity Status Close to Diagnosis and Its Association with Prostate Cancer Survival in the UK Biobank
Source: Cancer Res Commun. 2025 Jul 16;5(7):1155–70. doi: 10.1158/2767-9764.CRC-25-0124 (PMC12264726; doi:10.1158/2767-9764.CRC-25-0124)
Supplement: Supplementary Table 7 — Comparison of major lifestyle characteristics of men with BMI data according to the eligibility criteria of the present study versus those excluded because they did not have adiposity data and versus those included in the main models. [file crc-25-0124_supplementary_table_7_suppst7.docx]

| **Supplementary Table 7 – Comparison of major lifestyle characteristics of men with BMI data according to the eligibility criteria of the present study versus those excluded because they did not have adiposity data and versus those included in the main models.** | | | | |
| --- | --- | --- | --- | --- |
|  | Men excluded from the eligible sample of men with prostate cancer (N=8,619) because they did not meet eligibility criteria (i.e., no data on all the four adiposity indices and the covariates of the main model according to the eligibility criteria of the present manuscript). | Men with prostate cancer and adiposity data for all the four adiposity indices (BMI, hip circumference, waist circumference, waist-to-hip ratio) and on the covariates used in the main model age of diagnosis, year of diagnosis, smoking status, physical activity, sedentary activities, Townsend deprivation index and alcohol intake frequency. | | |
|  | N=4,859 | N=3,760  (pre/post-diagnosis) | N=2,370  (post-diagnosis) | N=1,390  (pre-diagnosis) |
| *Age at diagnosis, years, median (p2.5-p97.5)* | 66 (52-75) | 64 (52-71) | 63 (52-70) | 66 (54-72) |
| *Anthropometry (BMI baseline/at recruitment)* |  |  |  |  |
| BMI kg/m^2^, median *(p2.5- p97.5)* | 27 (21-36) | 27 (21-336) | 27 (22-36) | 27 (21-36) |
| Normal weight (≤24.9 kg/m^2^), n (%) **^a^** | 1179 (24) | 927 (25) | 563 (24) | 364 (26) |
| Overweight (25-29.9 kg/m^2^), n (%) | 2513 (52) | 1948 (52) | 1226 (52) | 722 (52) |
| Obese (≥30 kg/m^2^), n (%) | 1132 (23) | 885 (24) | 581 (25) | 304 (22) |
| Unknown, % (n) | 35 (0.7) | - | - | - |
| *Smoking status (at baseline)* |  |  |  |  |
| Never smoker, n (%) | 2285 (47) | 1789 (48) | 1153 (49) | 636 (46) |
| Current smoker, n (%) | 447 (9) | 303 (8) | 183 (8) | 120 (9) |
| Previous smoker, n (%) | 2053 (42) | 1668 (44) | 1034 (44) | 634 (46) |
| Unknown/missing, n (%) | 74 (2) | - | - | - |
| *Townsend deprivation index (at baseline)* **^b^** |  |  |  |  |
| *Townsend deprivation index (median p2.5-p97.5)* | -2.4 (-5.5 to 6.03) | -2.51 (-5.5 to 5.61) | -2.48 (-5.6 to 5.7) | -2.5 (-5.45-5.60) |
| Quintile 1 – least deprived | 971 (20) | 755 (20) | 474 (20) | 278 (20) |
| Quintile 2 | 970 (20) | 749 (20) | 475 (20) | 279 (20) |
| Quintile 3 | 971 (20) | 752 (20) | 473 (20) | 277 (20) |
| Quintile 4 | 970 (20) | 752 (20) | 474 (20) | 278 (20) |
| Quintile 5 – most deprived | 971 (20) | 752 (20) | 474 (20) | 278 (20) |
| Unknown/missing, n (%) | 6 (0.1) | - | - | - |
| *Physical activity (excess MET-hours/week), median (p2.5-p97.5) (at baseline)* | 22.1 (0.0-146.3) | 20.6 (0.0-136.0) | 19.6 (0-133.8) | 22 (0.0-138) |
| *±Sedentary activities (hours/day) median (p2.5-p97.5) (at baseline)* | 5 (2-11) | 5 (2-11) | 5 (2-11) | 5 (2-11) |
| *Alcohol intake frequency (at baseline)* |  |  |  |  |
| Never, n (%) | 287 (6) | 217 (6) | 149 (6) | 68 (5) |
| Special occasions only, n (%) | 359 (7) | 288 (8) | 191 (8) | 97 (7) |
| One to three times monthly, n (%) | 404 (8) | 305 (8) | 206 (9) | 99 (7) |
| Once or twice weekly, n (%) | 1197 (25) | 922 (25) | 583 (25) | 339 (24) |
| Three or four times weekly, n (%) | 1234 (25) | 956 (25) | 603 (25) | 353 (25) |
| Daily or almost daily, n (%) | 1355 (28) | 1072 (29) | 638 (27) | 434 (31) |
| Unknown/missing, n (%) | 23 (0.5) | - | - | - |
| *Ethnicity* **^c^** |  |  |  |  |
| White, n (%) | 4608 (95) | 3602 (96) | 2275 (96) | 1327 (95) |
| Other, n (%) | 251 (5) | 158 (4) | 95 (4) | 63 (5) |
| **^a^** The normal weight category includes the underweight as the number of men with underweight was very small (<10 men).  **^b^** Townsend deprivation index quintiles for men with prostate cancer but no adiposity data on all the four adiposity indices (BMI, waist circumference, hip circumference, waist-to-hip ratio) nor complete data on the covariates of interest, according to the eligibility criteria of this study, N=4,859, levels: [-6.26, -3.99] (-3.99, --2.91] (-2.91, -1.66] (-1.66,0.902] (0.902,9.76].  Individuals in the pre-/post-diagnosis BMI analysis, N=3,760, levels: [-6.26, -4.08] (-4.08, -3.02] (-3.02, -1.82] (-1.82,0.651] (0.651,8.89]. Individuals in the post-diagnosis BMI analysis, N=2,370, levels: [-6.26, -4.03] (-4.03, -2.99] (-2.99, -1.81] (-1.81,0.772] (0.772,8.59].  Individuals in the pre-diagnosis BMI analysis, N=1,390, levels: [-6.18, -4.16] (-4.16, -3.08] (-3.08, -1.84] (-1.84,0.508] (0.508,8.89].  **^c^** Ethnicity: “White” includes White, British, Irish, any other white background; “Other” includes Mixed, Asian or Asian British, Black or Black British, Chinese, other ethnic group, those who did not know/prefer not to answer and any unknown (unknown in non-participants only, N=15). Percentages rounded to the nearest whole number. | | | | |
