# Supplementary material for: Adiposity Status Close to Diagnosis and Its Association with Prostate Cancer Survival in the UK Biobank
Source: Cancer Res Commun. 2025 Jul 16;5(7):1155–70. doi: 10.1158/2767-9764.CRC-25-0124 (PMC12264726; doi:10.1158/2767-9764.CRC-25-0124)
Supplement: Supplementary Table 8 — Sub-group analysis by smoking for men with BMI close to prostate cancer diagnosis (pre- or post-diagnosis BMI combined). [file crc-25-0124_supplementary_table_8_suppst8.docx]

| **Supplementary Table 8 – Sub-group analysis by smoking for men with BMI close to prostate cancer diagnosis (pre- or post-diagnosis BMI combined).** | | | | | | | |
| --- | --- | --- | --- | --- | --- | --- | --- |
|  | ***N_e/_N_t_*** | **Never**  **smokers** | ***N_e/_N_t_*** | **Previous smokers** | ***N_e/_N_t_*** | **Current**  **smokers** | ***p-interaction^b^*** |
| **BMI (per 5 kg/m^2^)** |  | **HR^a^ (95% CI)** |  | **HR^a^ (95% CI)** |  | **HR^a^ (95% CI)** |  |
| *All-cause mortality* | 267/1,788 | 1.32 (1.13-1.54) | 326/1,688 | 1.41 (1.23-1.62) | 87/284 | 0.96 (0.70-1.31) |  |
|  |  |  |  |  |  |  | *0.29* |
| *Prostate cancer-specific mortality* | 129/1,788 | 1.40 (1.12-1.74) | 168/1,688 | 1.40 (1.15-1.71) | 34/284 | 0.79 (0.46-1.34) |  |
|  |  |  |  |  |  |  | *0.37* |
| **^a^** Models adjusted for: age of diagnosis, year of diagnosis, physical activity (continuous as sum of excess MET-hours/week of walking, moderate and vigorous activity), sedentary activities (continuous as sum of time spent watching TV, using a computer screen, or driving in hours/day), Townsend deprivation index (in quintiles within each subset) and alcohol intake frequency (categorical as: never, special occasions only, one to three times monthly, once or twice weekly, daily or almost daily).  Models stratified by UK Biobank centre.  **^b^** ANOVA, Wald test for the p-value.  Abbreviations: BMI, Body mass index; CI, Confidence Interval; HR, Hazard Ratio; N_e,_ number of events; N_t,_ total number of men with prostate cancer. | | | | | | | |
