# Supplementary material for: Adiposity Status Close to Diagnosis and Its Association with Prostate Cancer Survival in the UK Biobank
Source: Cancer Res Commun. 2025 Jul 16;5(7):1155–70. doi: 10.1158/2767-9764.CRC-25-0124 (PMC12264726; doi:10.1158/2767-9764.CRC-25-0124)
Supplement: Supplementary Table 9 — Cox Proportional HRs and 95%CIs for the linear association between adiposity after removing 1) the first year of follow-up and 2) the first two years of follow-up in relation to all-cause and prostate cancer-specific mortality (main/fully adjusted model). [file crc-25-0124_supplementary_table_9_suppst9.docx]

| **Supplementary Table 9 – Cox Proportional HRs and 95%CIs for the linear association between adiposity after removing 1) the first year of follow-up and 2) the first two years of follow-up in relation to all-cause and prostate cancer-specific mortality (main/fully adjusted model).** | | | | | |
| --- | --- | --- | --- | --- | --- |
|  |  |  | **All-cause**  **mortality** |  | **Prostate cancer-specific mortality** |
|  | **N_t_** | **N_e_** | **HR^a^ (95% CI)** | **N_e_** | **HR^a^ (95% CI)** |
| **BMI (per 5 kg/m^2^)** |  |  |  |  |  |
| Pre- or post-diagnosis combined |  |  |  |  |  |
| Main analysis (no removal of follow-up) | 3,760 | 680 | 1.30 (1.18-1.44) | 331 | 1.33 (1.15-1.52) |
| Removing the first year of follow-up | 3,732 | 658 | 1.31 (1.19-1.45) | 316 | 1.33 (1.15-1.54) |
| Removing the first two years of follow-up | 3,652 | 610 | 1.30 (1.18-1.44) | 284 | 1.29 (1.11-1.50) |
| Pre-diagnosis |  |  |  |  |  |
| Main analysis (no removal of follow-up) | 1,390 | 262 | 1.25 (1.05-1.48) | 124 | 1.18 (0.92-1.51) |
| Removing the first year of follow-up | 1,385 | 257 | 1.25 (1.06-1.49) | 121 | 1.21 (0.94-1.56) |
| Removing the first two years of follow-up | 1,370 | 242 | 1.26 (1.06-1.51) | 110 | 1.20 (0.92-1.57) |
| Post-diagnosis |  |  |  |  |  |
| Main analysis (no removal of follow-up) | 2,370 | 418 | 1.37 (1.21-1.54) | 207 | 1.43 (1.20-1.69) |
| Removing the first year of follow-up | 2,347 | 401 | 1.38 (1.22-1.56) | 195 | 1.41 (1.18-1.69) |
| Removing the first two years of follow-up | 2,282 | 368 | 1.36 (1.19-1.55) | 174 | 1.35 (1.11-1.63) |
| **Waist circumference (per 10 cm)** |  |  |  |  |  |
| Pre- or post-diagnosis combined |  |  |  |  |  |
| Main analysis (no removal of follow-up) | 3,760 | 680 | 1.28 (1.19-1.37) | 331 | 1.30 (1.18-1.44) |
| Removing the first year of follow-up | 3,732 | 658 | 1.28 (1.19-1.38) | 316 | 1.30 (1.17-1.44) |
| Removing the first two years of follow-up | 3,652 | 610 | 1.26 (1.17-1.36) | 284 | 1.25 (1.12-1.40) |
| Pre-diagnosis |  |  |  |  |  |
| Main analysis (no removal of follow-up) | 1,390 | 262 | 1.27 (1.12-1.44) | 124 | 1.21 (1.01-1.46) |
| Removing the first year of follow-up | 1,385 | 257 | 1.28 (1.13-1.45) | 121 | 1.31 (1.10-1.58) |
| Removing the first two years of follow-up | 1,370 | 242 | 1.28 (1.12-1.45) | 110 | 1.30 (1.07-1.57) |
| Post-diagnosis |  |  |  |  |  |
| Main analysis (no removal of follow-up) | 2,370 | 418 | 1.30 (1.19-1.42) | 207 | 1.32 (1.16-1.51) |
| Removing the first year of follow-up | 2,347 | 401 | 1.30 (1.18-1.42) | 195 | 1.30 (1.13-1.48) |
| Removing the first two years of follow-up | 2,282 | 368 | 1.27 (1.15-1.40) | 174 | 1.24 (1.07-1.44) |
| **Hip circumference (per 10 cm)** |  |  |  |  |  |
| Pre- or post-diagnosis combined |  |  |  |  |  |
| Main analysis (no removal of follow-up) | 3,760 | 680 | 1.34 (1.21-1.49) | 331 | 1.42 (1.23-1.65) |
| Removing the first year of follow-up | 3,732 | 658 | 1.16 (1.10-1.23) | 316 | 1.20 (1.11-1.30) |
| Removing the first two years of follow-up | 3,652 | 610 | 1.15 (1.09-1.22) | 284 | 1.18 (1.09-1.28) |
| Pre-diagnosis |  |  |  |  |  |
| Main analysis (no removal of follow-up) | 1,390 | 262 | 1.21 (1.01-1.46) | 124 | 1.29 (0.98-1.69) |
| Removing the first year of follow-up | 1,385 | 257 | 1.11 (1.01-1.22) | 121 | 1.16 (1.02-1.33) |
| Removing the first two years of follow-up | 1,370 | 242 | 1.10 (1.00-1.21) | 110 | 1.14 (0.99-1.31) |
| Post-diagnosis |  |  |  |  |  |
| Main analysis (no removal of follow-up) | 2,370 | 418 | 1.43 (1.26-1.62) | 207 | 1.49 (1.24-1.79) |
| Removing the first year of follow-up | 2,347 | 401 | 1.20 (1.12-1.28) | 195 | 1.22 (1.11-1.34) |
| Removing the first two years of follow-up | 2,282 | 368 | 1.19 (1.11-1.27) | 174 | 1.20 (1.08-1.33) |
| **Waist-to-hip ratio (per 0.1 unit)** |  |  |  |  |  |
| Pre- or post-diagnosis combined |  |  |  |  |  |
| Main analysis (no removal of follow-up) | 3,760 | 680 | 1.35 (1.20-1.53) | 331 | 1.34 (1.12-1.60) |
| Removing the first year of follow-up | 3,732 | 658 | 1.34 (1.19-1.52) | 316 | 1.30 (1.09-1.56) |
| Removing the first two years of follow-up | 3,652 | 610 | 1.31 (1.16-1.50) | 284 | 1.24 (1.02-1.51) |
| Pre-diagnosis |  |  |  |  |  |
| Main analysis (no removal of follow-up) | 1,390 | 262 | 1.49 (1.21-1.83) | 124 | 1.51 (1.11-2.03) |
| Removing the first year of follow-up | 1,385 | 257 | 1.49 (1.21-1.84) | 121 | 1.46 (1.08-1.99) |
| Removing the first two years of follow-up | 1,370 | 242 | 1.50 (1.21-1.86) | 110 | 1.49 (1.08-2.06) |
| Post-diagnosis |  |  |  |  |  |
| Main analysis (no removal of follow-up) | 2,370 | 418 | 1.30 (1.11-1.52) | 207 | 1.30 (1.03-1.63) |
| Removing the first year of follow-up | 2,347 | 401 | 1.28 (1.09-1.51) | 195 | 1.24 (0.99-1.57) |
| Removing the first two years of follow-up | 2,282 | 368 | 1.24 (1.05-1.47) | 174 | 1.14 (0.89-1.46) |
| **^a^** Models adjusted for: age of diagnosis, year of diagnosis, smoking status (categorical as: never, current, previous), physical activity (continuous as sum of excess MET-hours/week of walking, moderate and vigorous activity), sedentary activities (continuous as sum of time spent watching TV, using a computer screen, or driving in hours/day), Townsend deprivation index (in quintiles) and alcohol intake frequency (categorical as: never, special occasions only, one to three times monthly, once or twice weekly, daily or almost daily). Models stratified by UK Biobank centre. The date of each respective assessment visit (according to the period that the individual was selected from) was considered as the start of follow-up (entry-time). The date of death or censoring (31^st^ Dec 2020) was considered as the end of follow-up (exit-time).  Abbreviations: BMI, Body mass index; CI, Confidence Interval; HR, Hazard Ratio; N_e,_ number of events; N_t,_ total number of men with prostate cancer. | | | | | |
